# Data integration for prediction of weight loss in randomized controlled dietary trials

**DOI:** 10.1038/s41598-020-76097-z

**Published:** 2020-11-18

**Authors:** Rikke Linnemann Nielsen, Marianne Helenius, Sara L. Garcia, Henrik M. Roager, Derya Aytan-Aktug, Lea Benedicte Skov Hansen, Mads Vendelbo Lind, Josef K. Vogt, Marlene Danner Dalgaard, Martin I. Bahl, Cecilia Bang Jensen, Rasa Muktupavela, Christina Warinner, Vincent Aaskov, Rikke Gøbel, Mette Kristensen, Hanne Frøkiær, Morten H. Sparholt, Anders F. Christensen, Henrik Vestergaard, Torben Hansen, Karsten Kristiansen, Susanne Brix, Thomas Nordahl Petersen, Lotte Lauritzen, Tine Rask Licht, Oluf Pedersen, Ramneek Gupta

**Affiliations:** 1 Department of Health Technology, Technical University of Denmark, Kgs. Lyngby, 2800 Denmark; 2 Sino-Danish Center for Education and Research, University of Chinese Academy of Sciences, Beijing, China; 3 Department of Nutrition, Exercise and Sports, University of Copenhagen, Copenhagen, Denmark; 4 National Food Institute, Technical University of Denmark, Kgs. Lyngby, Denmark; 5 Department of Anthropology, Harvard University, Cambridge, 02138 USA; 6 The Novo Nordisk Foundation Center for Basic Metabolic Research, Faculty of Health and Medical Sciences, University of Copenhagen, Copenhagen, 2200 Denmark; 7 Institute for Veterinary and Animal Sciences, University of Copenhagen, Frederiksberg, Denmark; 8 Department of Radiology, Bispebjerg Hospital, Copenhagen, Denmark; 9 Department of Medicine, Bornholms Hospital, Rønne, Denmark; 10 Laboratory of Genomics and Molecular Biomedicine, Department of Biology, University of Copenhagen, 2100 Copenhagen, Denmark; 11 Department of Biotechnology and Biomedicine, Technical University of Denmark, Kgs. Lyngby, Denmark; 12 Present Address: Novo Nordisk Research Centre Oxford, Oxford, OX3 7FZ UK

**Keywords:** Weight management, DNA, Metabolomics, Computational models, Data integration, Data processing, Machine learning, Predictive medicine, Genotype, Microbial genetics, Sequencing, Computer modelling, Computer science

## Abstract

Diet is an important component in weight management strategies, but heterogeneous responses to the same diet make it difficult to foresee individual weight-loss outcomes. Omics-based technologies now allow for analysis of multiple factors for weight loss prediction at the individual level. Here, we classify weight loss responders (N = 106) and non-responders (N = 97) of overweight non-diabetic middle-aged Danes to two earlier reported dietary trials over 8 weeks. Random forest models integrated gut microbiome, host genetics, urine metabolome, measures of physiology and anthropometrics measured prior to any dietary intervention to identify individual predisposing features of weight loss in combination with diet. The most predictive models for weight loss included features of diet, gut bacterial species and urine metabolites (ROC-AUC: 0.84–0.88) compared to a diet-only model (ROC-AUC: 0.62). A model ensemble integrating multi-omics identified 64% of the non-responders with 80% confidence. Such models will be useful to assist in selecting appropriate weight management strategies, as individual predisposition to diet response varies.

## Introduction

There is considerable interest in identifying markers that can predict responsiveness to various weight loss interventions^1^. Weight loss modelling has previously focused on energy intake and expenditure^2,3^, macronutrient balance^4^, anthropometrics^5^, glycemic and insulinemic statuses^6,7^ and gut microbiome profiles by the *Prevotella*-to-*Bacteroides* ratio^8^.

Multi-omics data has shown promise in improving the understanding of complex phenotypes such as metabolic heath^9,10^, which reflects an interplay between physiology, genome and exposome (diet, microbiome, metabolome) of a given individual. At the cohort level, associations to obesity have been found in the human gut microbiome^11^, the plasma metabolome^12^ and the host genome^13^. Integration of multiple omics has recently been applied for unravelling weight changes in insulin sensitive and insulin resistant individuals^14,15^. Results from these studies show progress towards signatures of weight loss, although inter-individual heterogeneity still leaves a challenge in individual level predictions. In general, computational integration of multi-omics data is challenging due to data heterogeneity, a large number of variables, small sample sizes and missing data^16^. Machine learning methods have shown some progress in this area, especially when coupled with adequate data handling and relevant feature reduction strategies^10,17^ and have been applied in prediction of the personal glycemic response to diet^18–20^. Further, ensemble methodologies have demonstrated improved stability on machine learning predictors^16,21^.

We previously investigated the impact of 8 weeks dietary interventions on human metabolic health outcomes in two Danish randomized cross-over trials with a whole grain-rich diet or low-gluten diet, associated with a beneficial and non-beneficial impact on metabolic health, respectively^22–24^, and identified weight loss as a response to each of the interventions relative to a refined grain diet^25,26^. It has however been argued that no single dietary strategy would be appropriate for all individuals and that certain biomarkers can be important in relation to predisposition for weight loss^7^. This study investigates the use of machine learning to predict which individuals who will experience weight loss during the 8 weeks of dietary interventions with whole grain, low gluten or refined grain. We present random forest-based data integration of anthropometry, blood serum markers, gut microbiome markers, urine metabolomics and host genomics to investigate, if the weight loss response can be predicted based on randomisation onto dietary intervention and biomarkers at baseline prior to any dietary intervention. Models were guided by prior knowledge as well as data-driven feature selection and representation strategies to improve predictability with limited cohort sizes (N = 102 participants across two intervention baselines). Performance and robustness were estimated through cross-validation and shuffling cross-validation sets, respectively. By identifying the propensity of study participants likely to experience weight loss, a more effective individual targeting of dietary interventions can be facilitated, eventually in concert with comprehensive population weight loss strategies. Furthermore, understanding predictive features of weight loss response will drive improved understanding of the interplay between gut microbiota, diet and individual predisposition.

## Results

### Personal weight loss response to whole grain-rich, low-gluten and refined grain diets

**Figure 1:**
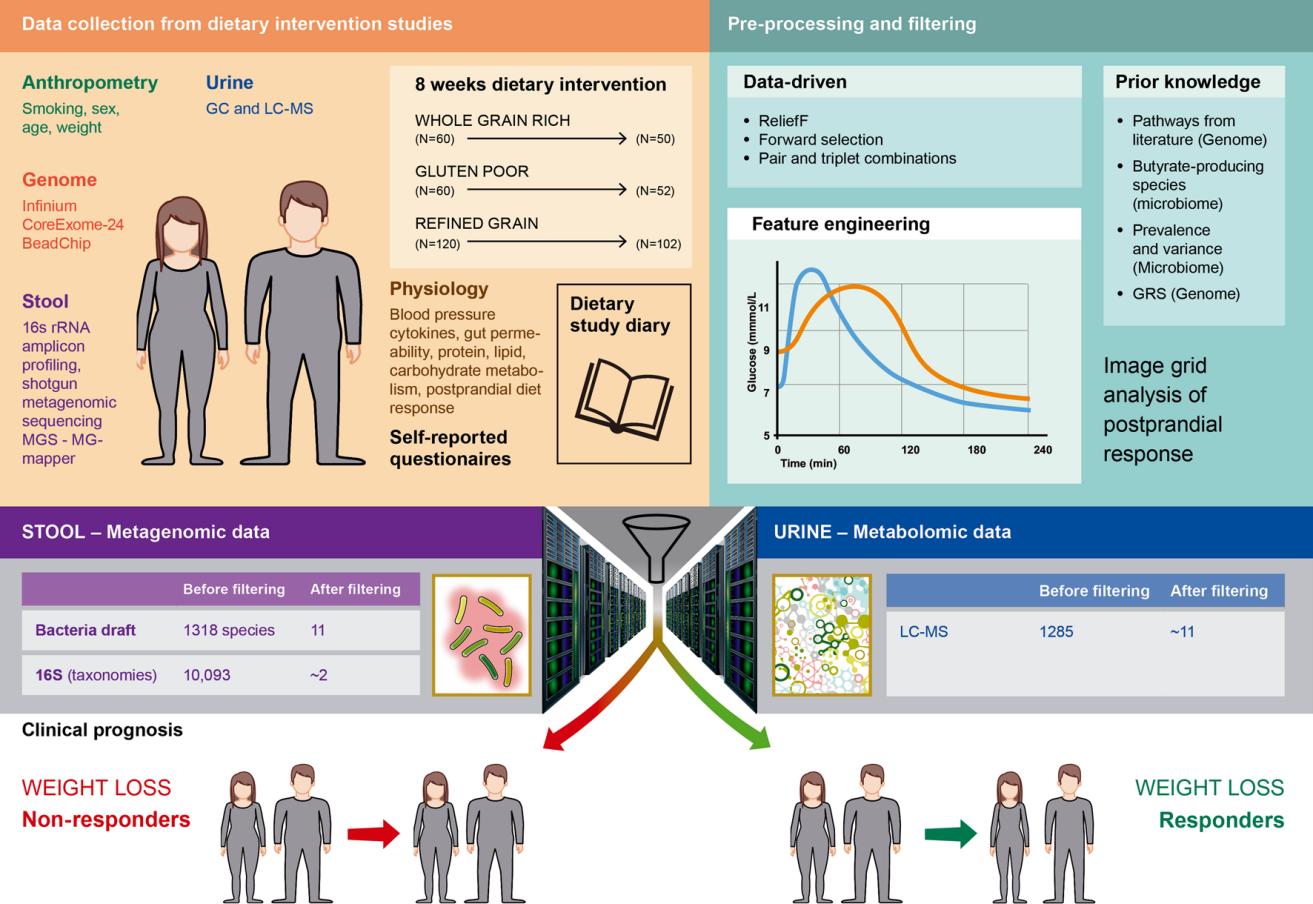
Study design including data availability, feature development and selection, best features selected for model and clinical prognosis of weight loss. Participants achieving any weight loss during 8 weeks dietary intervention were considered weight loss responders. Different combinations of features were selected for modelling e.g. included the faecal stool samples by butyrate-producing species from MGmapper catalog Bacteria draft and by forward selected 16S taxonomies selected from a pool of the top 250 most varying. These were combined with forward selected urine metabolites identified by LC–MS. Only measurements from the beginning of the intervention periods were used as features for development of predictive weight loss models.

**Figure 2:**
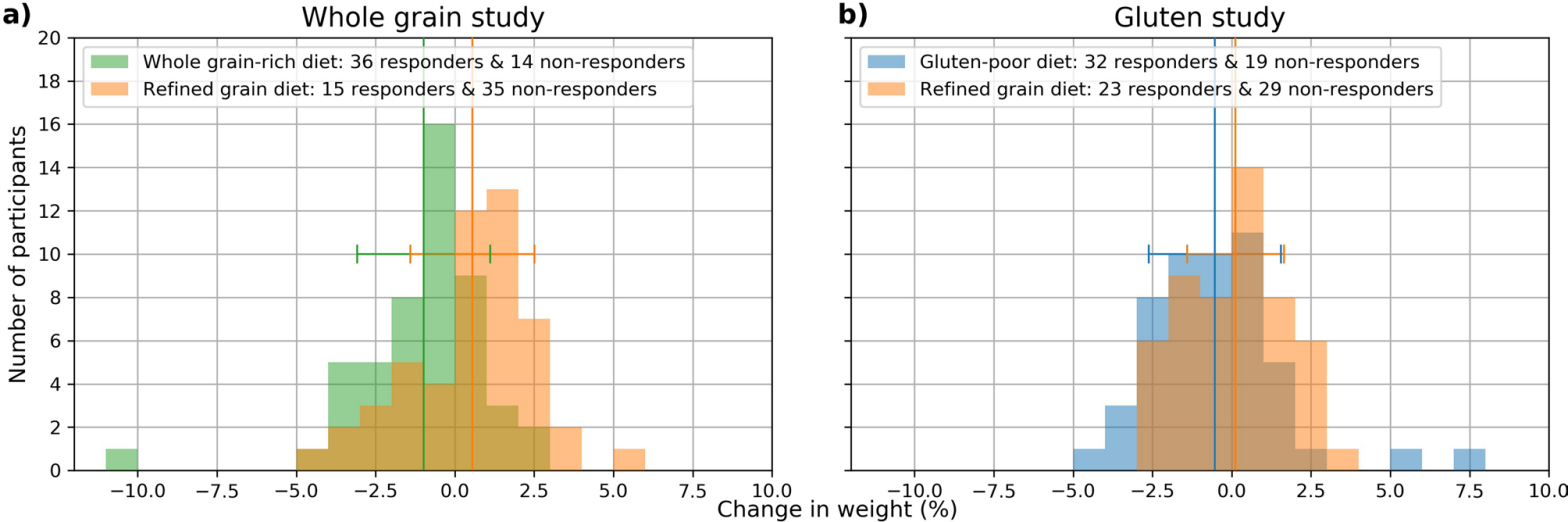
Weight changes in the two dietary intervention arms during 8 weeks (**a**) Distribution of percentage changes in body weight for the whole grain study. (**b**) Distribution of percentage changes in body weight for the gluten study. The coloured lines denote mean and standard deviations for the diet groups (green = whole grain-rich diet, orange = refined grain diet, blue = low-gluten diet).

In our previous whole grain diet study, 60 study participants with a cardiometabolic risk profile were randomized to follow either a whole grain-rich diet or a refined grain diet for 8 weeks before cross-over to the other study diet after a 6 weeks wash-out period^25^. In a similarly designed study, also with sixty study participants, a low-gluten diet was compared to the same refined grain diet^26^, which was designed to have high gluten and low whole grain content. An overview of the study design and collection of data is shown in Fig. 1. The participants were examined before and after each of the dietary intervention periods (whole grain-rich diet, low-gluten diet or refined grain diet), where data was collected on anthropometrics, physiology, urine metabolites, gastrointestinal transit time, faecal stool samples for gut microbiome analyses and host genomics. In total, 102 participants completed the whole grain (N = 50 participants) and gluten (N = 52 participants) clinical trials. Both trials reported significant weight loss following each of the interventions compared to a refined grain diet^25,26^. However, weight loss or gain was observed across all three dietary interventions (Fig. 2a for the whole grain trial and Fig. 2b for the gluten trial). In this study, we classified weight loss responders (N = 106 individuals) or non-responders (N = 97 individuals) across the dietary interventions by machine learning-modelling of biomarkers measured at the start of the intervention periods (baseline) where given individual biomarkers may predispose weight loss on a given dietary intervention. Each individual weight change during the interventions was considered (N = 204 individuals). One individual in the gluten trial had missing values of body weight in an intervention and was excluded from the analysis. Thus, 203 individuals were used to model body weight response given the dietary interventions. An individual with any degree of weight loss after the 8-week intervention compared to baseline body weight was considered a responder (range: − 0.06 to − 10.43%), whereas no change or weight gainers were classified as non-responders independent of the dietary study arm.

In the whole grain-rich diet intervention, low-gluten diet intervention or refined grain diet intervention, study participants experienced relative body weight changes ranging between $\pm \;5\%$ after 8 weeks of dietary changes relative to their baseline body weight. Weight loss responders experienced a relative body weight decrease of on average 1.67% $\pm$ 1.42%, while weight gain non-responders had an average relative body weight increase of 1.39% $\pm$ 1.2%.

### Predictability of weight loss using diet information alone

Random forests were used to develop predictive models of weight loss responders and non-responders, where we ensured that the same model initialization across 50 shuffle-split fivefold cross-validations was used (Supplementary Material 1). This machine learning setup was used for all trained models reported in this paper. A baseline performance of the area under the receiver operating characteristic curve (ROC-AUC): 0.62 was established (N = 203 individuals) using information only about the type of diet. Inclusion of the accurate continuous whole grain intake (2.0–210.28 g/day) and gluten intake (3193.85–22,961.47 mg/day) at baseline as a potential predictor of weight loss together with the type of diet resulted in ROC-AUC: 0.63 (N = 201 individuals). Thus, the type of dietary intervention and the habitual whole grain and gluten intake is not sufficient to predict weight loss for all individuals during the dietary intervention.

### Prior knowledge feature development for machine learning-based data integration

We integrated information about heterogeneous biomarkers of metabolic health into machine learning models in order to understand the individual level omics and physiological predisposition profiles to weight loss using measurements at the baseline intervention only. We had in total 287,596 features available for modelling (Table 1, in Supplementary File 1, the exact features available for modelling for each dataset and features selected by forward selection are given. Changes in biomarkers during the interventions are given in Supplementary File 2); 28 anthropometric and physiological features (Clinical), gastrointestinal transit time (TransitTime), 10,093 16S-based OTUs (16S), 3490 shotgun sequenced species (mapped to taxa by MGmapper (MGm) or as metagenomic species (MGS)), 1370 urinary metabolites (analysed by gas-chromatography mass spectrometry (GC–MS) and liquid chromatography mass spectrometry (LC–MS)) and 272,588 single nucleotide polymorphisms (SNPs) from the host genome (LithPath, LithPathLD and GRS).

**Table 1:** Overview of datasets, number of features and feature selection for random forest models.

Data type	Data label	Number of features before filtering	Number of features after prior knowledge filtering	Was data-driven feature selection applied (Y/N)
Diet: Binary features that represent the type of diet as whole grain-rich, low-gluten or refined grain	Diet	3	–	N
Anthropometrics and physiological	ClinicalA	28	8 (age, sex, BMI and blood CRP, IL-6, HbA1c, HOMA-IR and zonulin)	N
	ClinicalB		–	Y
Whole grain and gluten intake	ContinuousIntake	2	–	N
Gastrointestinal transit time	TransitTime	1	–	N
Self-reported	VAS	16	–	N
Postprandial response	PostPran	5	–	N
**Genome data**		272,588 SNPs (after QC)		
Literature pathways	LitPath		703 SNPs	Y
LD pruned literature pathways	LitPathLD		56 SNPs	Y
Genetic risk scores	GRS		5 GRS’s of 32 SNPs	N
**Metagenomic data**				
16S (taxonomies)		10,093		
Top 10 most variating	16S_A		10	N
Top 250 most variating	16S_B		250	Y
Prevalence	16S_C		3321	Y
**MGmapper (species)**				
Bacteria catalogue	MGm_A	464	9	N
Bacteria draft catalogue	MGm_B	1318	–	Y
	MGm_B1		11	N
Human microbiome catalogue	MGm_C	444	10	N
Butyrate-producing species from MGmapper catalogues	MGm	–	30	N
Metagenomic species	MGS	1264	–	Y
Top 14 from whole grain and gluten studies	topMGS		28	N
**Metabolic data**				
GC–MS	GC–MS	85	–	Y
LC–MS	LC–MS	1285	–	Y

To guide the machine learning models, we developed features and prioritised biomarkers for modelling by prior knowledge strategies (detailed information is given in “Methods”). We therefore ended up with eight clinical variables of glucose metabolism, inflammation, gut permeability and anthropometric traits (ClinicalA), 703 SNPs annotated to genes involved in selected metabolic pathways, inflammation and gut microbiome composition identified in pertinent literature (LithPath and LithPathLD, Supplementary Material 2a), 250 most varying 16S-based OTUs during the dietary interventions (16S_B) and 30 shotgun sequenced faecal microbiome species features (mapped by MGmapper to butyrate-producing species (MGm and MGm_ABC, Supplementary Material 2b). We also considered information from the changes in MGS’ in the previous clinical trial studies^25,26^, where the changes in the relative abundance of MGS’ when on refined grain diet was compared to a whole grain-rich diet or low-gluten diet. For the gluten study, 14 MGS’ were found significantly changing in abundance^26^, when comparing the changes in abundance for the two dietary interventions. No MGS’ changed significantly in the whole grain study^25^. From both studies, the top 14 most significant MGS’ were included for modelling of weight loss (topMGS, Supplementary Material 2c). In addition, we developed five genetic risk scores (GRS); three for obesity phenotypes defined by BMI from literature, one for body weight change and one for sagittal abdominal diameter change after the whole grain intervention compared to the refined grain diet intervention (Supplementary Material 2d). Finally, we modelled features of the longitudinal measurements of the postprandial response including breath hydrogen and plasma free fatty acids, GLP-2, glucose and insulin by an image analysis approach of the postprandial dynamics to capture volatility in addition to the AUC (PostPran).

### Weight loss prediction is improved by inclusion of gut microbiome and urinary metabolome features

To identify metabolic profiles predictors of weight loss following a whole grain-rich, low-gluten or refined grain dietary intervention, we tested the predictive performance of each data type separately and ensured that the same study participants were available across 18 out of 22 datasets to allow for comparison of models performance (N = 130 individuals; 63 non-responders and 67 responders, referred to as complete data models). All complete data models included information of which type of diet the study participants were randomised to receive, since data from both baselines (start of intervention periods) in the cross-over studies were used for modelling of weight loss responders and non-responders. On this subset, information only about diet type gave a ROC-AUC: 0.61 (Diet in Table 2). Several models were trained by adding features to this model, where feature sets included all available features for a given data type, as well as the prior knowledge-developed datasets with and without data-driven feature selection methods (see all combinations for complete data models in Supplementary Material 3 and selected reported combinations in Table 2). The most predictive models were identified by data-driven selection of microbiome signatures of MGmapper species and top 250 most varying 16S-based OTUs when considering each type of biological information separately (Table 2; Diet.MGm_B with ROC-AUC: 0.82 and Diet.16S_B with ROC-AUC: 0.82, respectively). To explore the predictive signals identified in the intestinal gut microbiome features further, we integrated gut microbiome signatures from 16S-based OTUs from a pool of the top 250 species most varying during the dietary interventions (16S_B) or butyrate-producing species from the MGmapper Bacteria draft database (MGm_B1) along with urine metabolites identified by LC–MS (LC–MS). For the model Diet.16S_B.LC–MS, the type of diet was always included, while we forward selected features from the 16S-based OTUs and the LC–MS together. For the model Diet.MGm_B1.LC–MS, we only forward selected on the urine metabolites identified by LC–MS, while the type of diet and butyrate-producing species from the MGmapper Bacteria draft database always were included. This resulted in the best performing models for weight loss predictions with performance of ROC-AUC: 0.86 and ROC-AUC: 0.90, respectively (Diet.16S_B.LC–MS and Diet.MGm_B1.LC–MS in Table 2).

**Table 2:**
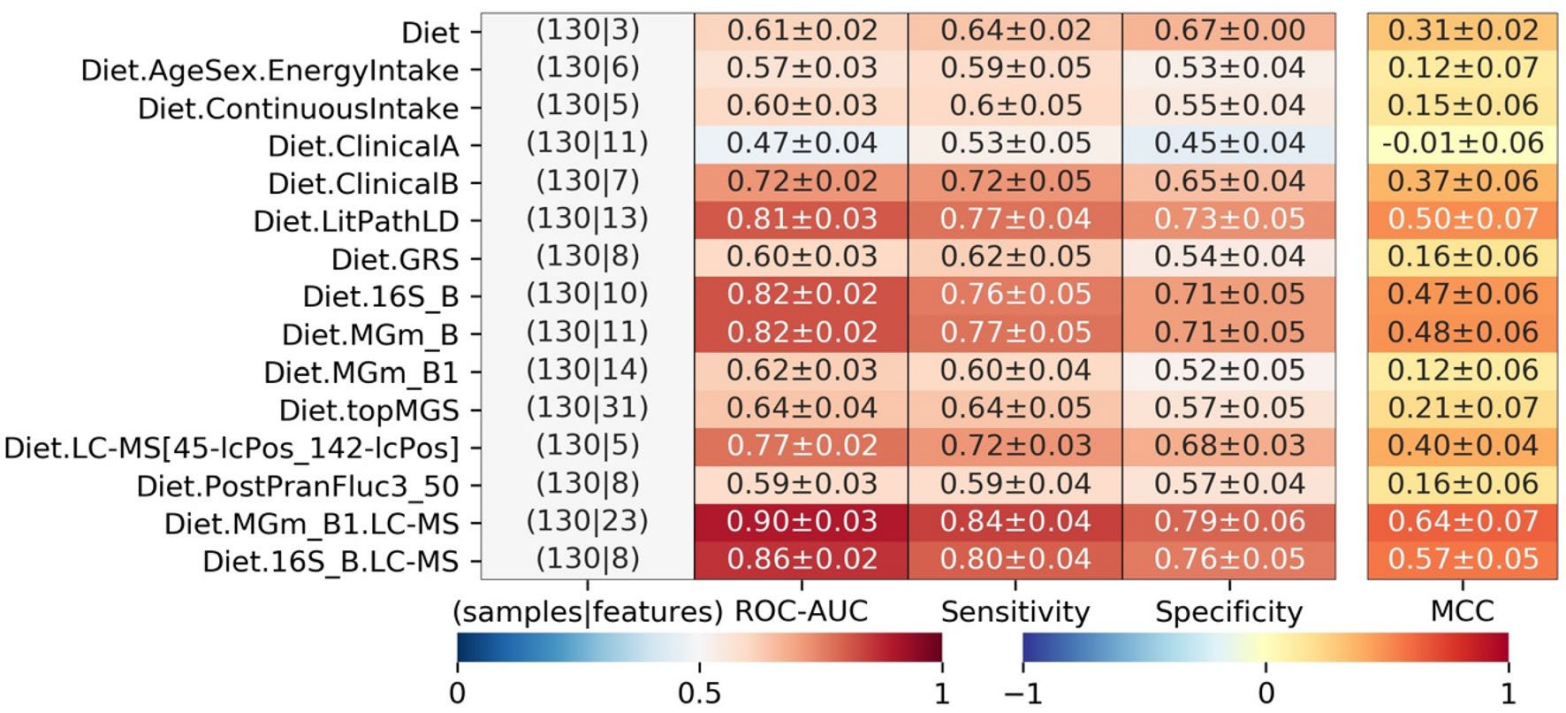
Model performances for models run on a set of 130 individuals with complete data in all below data combinations.

This is reported as mean of five cross-validations repeated 50 times with random shuffles of the cross-validation splits. The blue-red colorbar is for area under the receiver operating characteristic curve (ROC-AUC), sensitivity and specificity, while the blue-yellow–red colorbar is for Matthews correlation coefficient (MCC). Diet represents the dataset consisting of the three features indicating which diet was consumed. EnergyIntake is the energy intake at baseline, while ContinuousIntake is the total intake of whole grain (g/day) and gluten (mg/day) at baseline. ClinicalA and B are both feature subsets selected by prior knowledge and forward selection, respectively, from the set of 28 anthropometric and physiological features. LithPathLD and GRS are subsets of genetic variants selected by prior knowledge, where LithPathLD also was subject to forward selection. 16S_B is the set of forward selected 16S-based OTUs selected from a pool of the top 250 most varying features. MGm_B and MGm_B1 are subsets of species mapped by MGmapper to the Bacteria draft catalogue, which are selected by forward selection and prior knowledge as butyrate-producing species, respectively. LC–MS[45-lcPos_142-lcPos] holds a pair of urine metabolites identified by LC–MS. PostPranFluc3_50 is the post prandial response features free fatty acids, GLP-2, glucose and insulin, which are represented by the third image analysis method with a grid-size of 50 × 50 (see “Methods”). Abbreviations for model combinations are explained in Table 1 and in the main text. Performances of all models run on the 130 individuals are in Supplementary Material 3.

We ensured that models were not overfitted by performing a permutation analysis on the prediction outcome of weight loss responders or non-responders for a total of 50 times. The model robustness was assessed using a randomly permuted prediction class label where models were allowed to retrain using the features selected by the model trained on the true prediction class label. Models trained on the true prediction class label performed significantly better than a randomly permuted label given the most predictive features; p <  < 1 * 10^–6^, Supplementary Material 4, Figure S.5A (see also Figures S.5B and S.5C for other permutation approaches).

### Microbiome and metabolome association to weight loss

After establishing that random forest models including features of the faecal microbiome, urine metabolome and the type of diet (whole grain-rich, low-gluten or refined grain) were most predictive of weight loss, we expanded the random forest models to include all samples that were available for each given data combination (N = 147–203 individuals; 74–97 non-responders and 73–106 responders depending on data type, Table 3; all trained models in Supplementary Material 5). The best performing models for weight loss predictions were again Diet.16S_B.LC–MS (ROC-AUC: 0.84, N = 169 individuals) and Diet.MGm_B1.LC–MS (ROC-AUC: 0.88, N = 173 individuals).

**Table 3:**
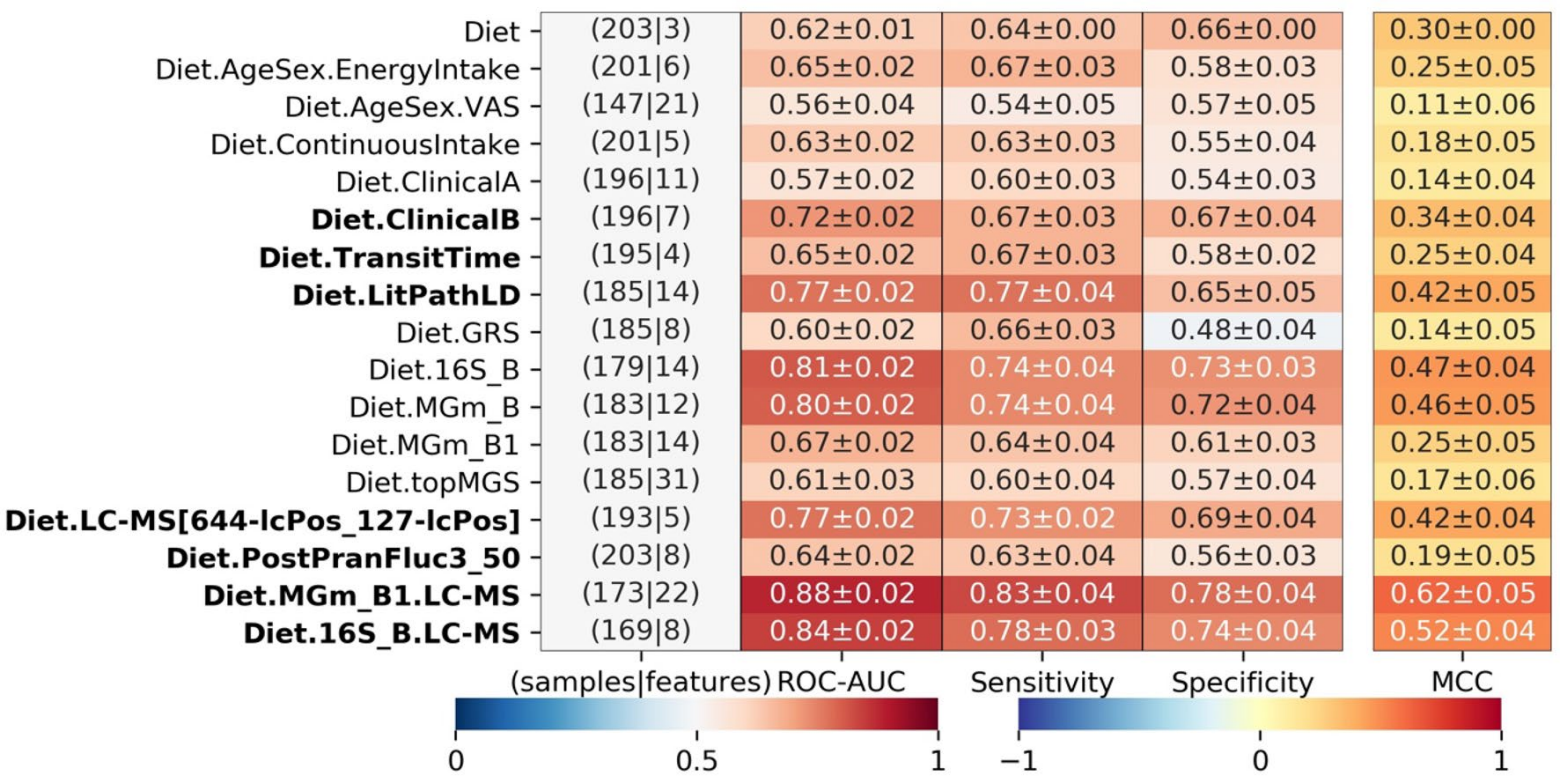
Model performances for models run on all individuals available for a given data combination.

This is reported as mean of five cross-validations repeated 50 times with random shuffles of the cross-validation splits. Models in bold were included in an ensemble (ROC-AUC > 0.62). The blue-red colorbar is for area under the receiver operating characteristic curve (ROC-AUC), sensitivity and specificity, while the blue-yellow–red colorbar is for Matthews correlation coefficient (MCC). Diet represents the dataset consisting of the three features indicating which diet was consumed. EnergyIntake is the energy intake at baseline, while ContinuousIntake is the total intake of whole grain (g/day) and gluten (mg/day) at baseline. VAS represents the self-reported features measured by Visual Analogue Scale. ClinicalA and ClinicalB are both feature subsets selected by prior knowledge and forward selection, respectively, from the set of 28 anthropometric and physiological features. TransitTime is the baseline transit time. LithPathLD and GRS are subsets of genetic variants selected by prior knowledge, where LithPathLD also was subject to forward selection. 16S_B is the set of forward selected 16S-based OTUs selected from a pool of the top 250 most varying features. MGm_B and MGm_B1 are subsets of species mapped by MGmapper to the Bacteria draft catalogue, which are selected by forward selection and prior knowledge as butyrate-producing species, respectively. topMGS is the top 28 selected MGSs from the whole grain and gluten studies. LC–MS[45-lcPos_142-lcPos] holds a pair of urine metabolites identified by LC–MS. PostPranFluc3_50 is the post prandial response features free fatty acids, GLP-2, glucose and insulin, which are represented by the third image analysis method with a grid-size of 50 × 50 (see “Methods”). Abbreviations for model combinations is explained in Table 1 and in main text. Performances of all models run are in Supplementary Material 5.

**Figure 3:**
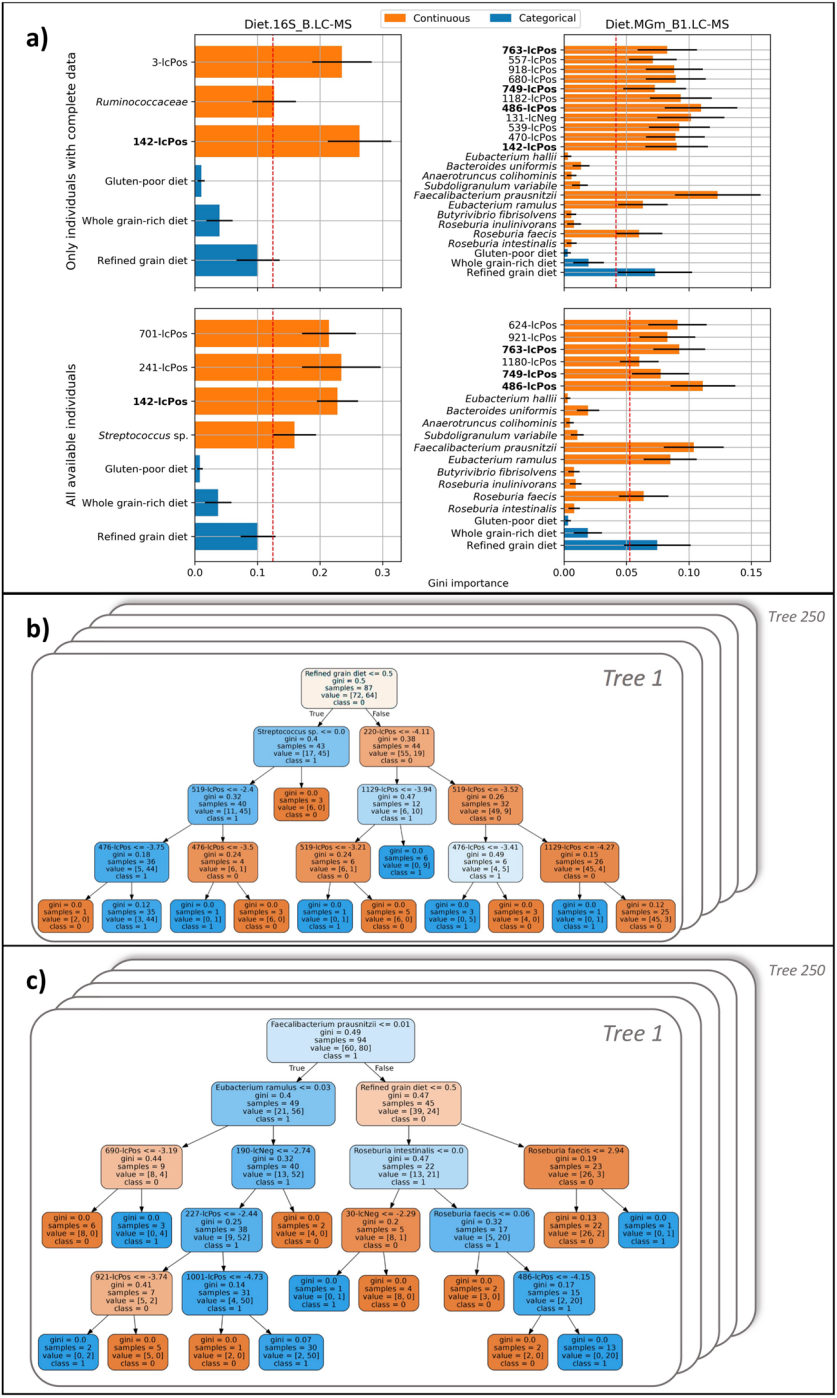
Feature importance for the models. (**a**) Models have data combinations of the type of diet, forward selected 16S-based OTUs from a pool of the top 250 most varying (left, Diet.16S_B.LC–MS) or butyrate-producing species (right, Diet.MGm_B1.LC–MS) and forward selected urine metabolites identified by LC–MS for features selected minimum 15% across all trained models. The columns represent the two data combinations, and the rows represent the runs on 130 common individuals with complete data across 18 out of 22 datasets (*Only individuals with complete data*) as well as runs on all individuals available for the data combination (*All available individuals*). The red line marks features of highest importance given the relative Gini coefficient. (**b**,**c**) Illustrations of the random forest models trained and tested on diet, forward selected 16S-based OTUs from a pool of the top 250 most varying (**b**, Diet.16S_B.LC–MS) or butyrate-producing species (**c**, Diet.MGm_B1.LC–MS) and forward selected urine metabolites identified by LC–MS with all available individuals. The labels on the tree leaves represent per leaf the gini impurity, the number of unique individuals, the distribution of classes for the bootstrap sample and the class which holds majority. The colours denote the class which holds majority as well as magnitude of majority by more saturation, where orange colour is the non-responders (class 0) and blue is the responders (class 1).

The feature importance, represented by the Gini coefficient in the random forest models, was reported for the selected intestinal microbiome features (16S-based OTUs or butyrate-producing species from MGmapper species) and the urinary metabolomic features in the four best random forest models [two models trained on N = 130 individuals with complete data for comparison of models, and two models on N = 169/173 individuals for models including all available individuals for the data type combination (Fig. 3)].

For forward selected features, only features selected in at least 15% of all trained random forest models across the 50 shuffle-split fivefold cross-validations are reported. In all four models, the type of diet was considered important from evaluation of the Gini coefficient (above the red line in Fig. 3a). The main impact for classification related to whether study participants received a refined grain diet, whole grain-rich or low-gluten diet, seen as the refined grain diet was considered most important by all models (Fig. 3a). This was expected, as a statistically significant relative weight loss was previously found after consuming the whole grain-rich or the low-gluten diet compared to the refined grain diet in the two clinical trials^25,26^. All types of diet were considered equally in the training of the random forest models. Summary statistics between responders and non-responders as well as putative annotations for most important urinary metabolite features identified by LC–MS and microbiome features are provided in Supplementary Material 6. Metabolites were selected through a data-driven forward selection approach from a total of 1285 urinary metabolites identified by LC–MS. In the two models of Diet.16S_B.LC–MS*,* the taxonomies for the forward selected microbial species were from a pool of the 250 most varying 16S-based OTUs in the intervention periods together with urine metabolites identified by LC–MS. Only the family *Ruminococcaceae* and genus *Streptococcus* were selected in enough models (15%) to be considered important given the number of all selected features as seen in Fig. 3a (left column) (ROC-AUC: 0.86 for N = 130 individuals and ROC-AUC: 0.86 for N = 169 individuals, Tables 2 and 3). *Ruminococcaceae* was most abundant in weight loss responders. *Streptococcus* was, by contrast, more abundant in non-responders. The species in the two Diet.MGm_B1.LC–MS models were pre-selected based on literature extracted butyrate-producing species (Supplementary Material 2a) and have been identified as being important for metabolic health, where the species *Faecalibacterium prausnitzii, Eubacterium ramulus* and *Roseburia faecis* are of special importance to the model with a selected range of urine metabolites as seen in Fig. 3a (right column) (ROC-AUC: 0.90 for N = 130 individuals and ROC-AUC: 0.88 for N = 173 individuals, Tables 2 and 3).

### An ensemble of multi-omics models is more robust to varying input data

We explored if the combination of multiple trained weight loss prediction models could improve prediction performance. Further, as given individuals had different missing data, the ensemble approach allows use of all available omics data. The ensemble was made from a selection of the different combinations of potential prediction models performing ROC-AUC > 0.62 (Diet baseline performance for all available models (N = 203 individuals)). This approach resulted in an ensemble consisting of 334 out of a total 350 models across seven different input data combinations and 50 shuffle-split cross-validations. The seven data combinations include features of diet, forward selected clinical features, SNPs annotated to genes in metabolic pathways, inflammation and gut microbiome composition identified from a literature search, post-prandial response, gastrointestinal transit time, butyrate-producing species, 16S-based OTUs and urinary metabolites identified by LC–MS. All included models are marked in bold in Table 3.

**Figure 4:**
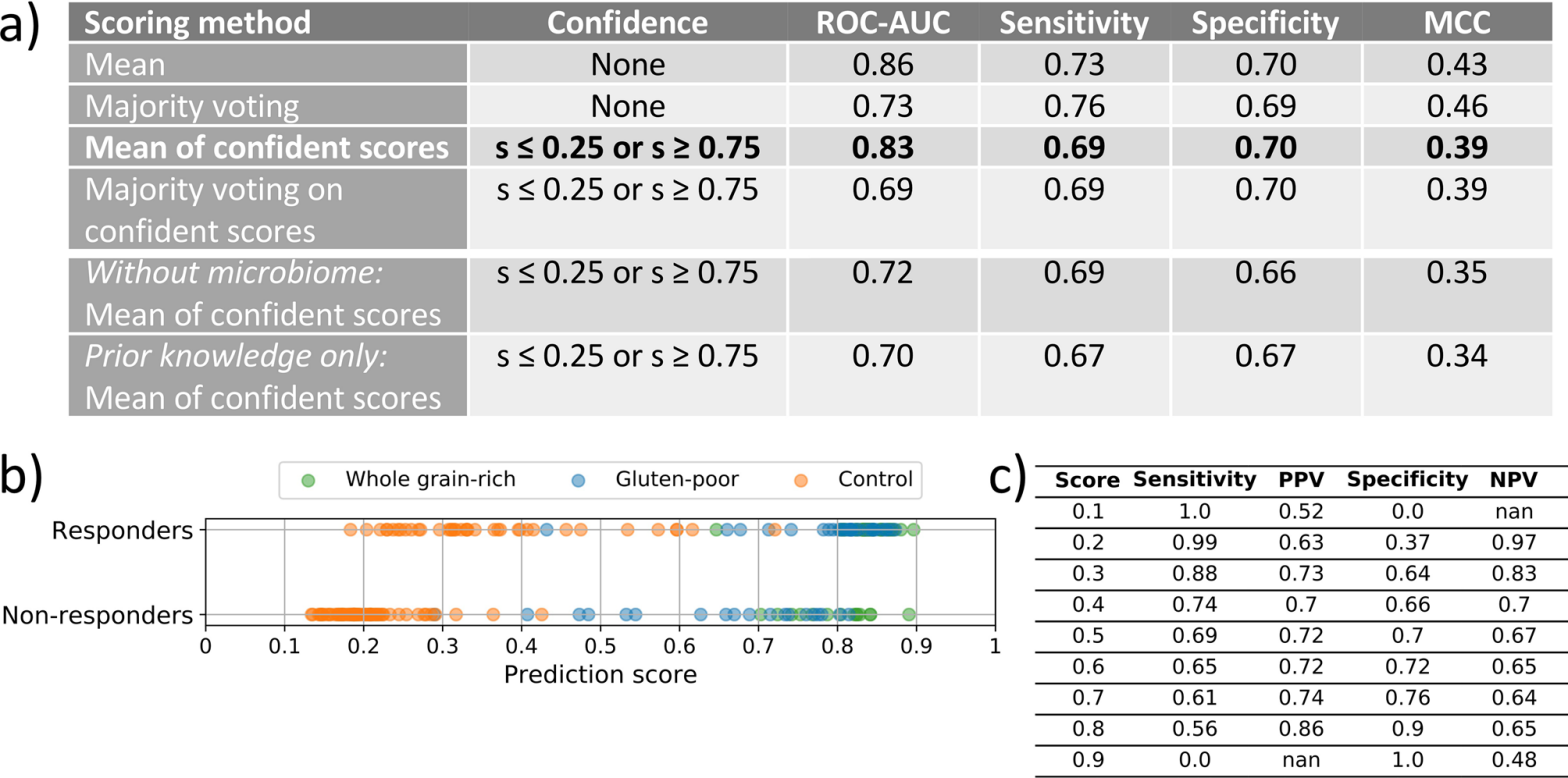
Ensemble of weight loss models. (**a**) Performances based on four scoring schemes and different classification thresholds for predictive models included in different personalised ensemble models. The confidence column shows the applied prediction score thresholds, where *s* is the prediction score. The first four rows are the ensemble presented consisting of models in bold in Table 3, where an extension of this to other confidence thresholds is found in Supplementary Material 7, Table S.6. The ensemble in bold is represented in (**b**,**c**). *Without microbiome* is an ensemble of same models as in bold in Table 3 but excluding all models that contain microbiome data. *Prior knowledge only* is an ensemble of models that only include features selected by prior knowledge feature selection approaches. (**b**) The prediction scores across responders or non-responders with colors representing the type of dietary intervention. The scores shown are from ensemble scoring method mean of confident scores (s ≤ 0.25 or s ≥ 0.75). (**c**) The sensitivity, positive predictive value (PPV), specificity and negative predictive value (NPV) are calculated at different score thresholds to separate the classes for the ensemble model shown in (**b**). MCC: Matthews correlation coefficient.

The ensemble model was evaluated using different scoring methods to make a combined prediction score, *s*, per individual. These scores range 0 to 1, where 0 is the non-responder class and 1 is the responder class. The highest performing ensemble achieved ROC-AUC: 0.86 by averaging prediction scores from the seven original models, while performances of other ensemble models that include predictions of a sufficiently high confidence ranges in ROC-AUC from 0.69 to 0.84 (Fig. 4a). The best ensemble model thus performs very similar to the Diet.16S_B.LC–MS (ROC-AUC: 0.84) and Diet.MGm_B1.LC–MS (ROC-AUC: 0.88) models.

The fully integrated ensemble model based on the mean of confident scores (s ≤ 0.25 or s ≥ 0.75) was chosen as a final model to only allow for confident predictions to make a final count in the individual weight loss prediction. In order to only identify highly confident weight loss responders or non-responders, the prediction score threshold that divides the classes was varied for the models included in the ensemble (Fig. 4b,c). Using s = 0.30 as the classification threshold, the ensemble model correctly classified 64% of all individuals who gained or maintained body weight on a given diet (non-responders) where only 17% of these were false negative classifications (responders to diet). Conversely, by setting a threshold of s = 0.70 for detection of people who will experience a weight loss, the ensemble model predicted 61% of the individuals at a cost of 26% false positive predictions. Scoring the ensemble model only with highly confident predictions (s ≤ 0.25 or s ≥ 0.75) excluding models using the gut microbiome and the urine metabolome, resulted in ROC-AUC: 0.72 (*Without microbiome*, Fig. 4a). Therefore, clinical information, host genotype, the post-prandial response features and gastrointestinal transit time should be considered important in weight loss predictions as well, if the information of gut microbiome and urinary metabolites is not available when deploying the model.

## Discussion

We investigated if it was possible to predict weight loss responders and non-responders following specific dietary interventions over a period of 8 weeks in healthy Danish subjects with a cardio-metabolic risk profile. Both dietary trials previously reported significant weight loss after the intervention with whole grain-rich and low-gluten diets, respectively, compared to a refined grain diet with the use of linear mixed models^25,26^. Despite the overall statistical significance of weight loss, the individuals differed widely, and weight loss was reported on all three dietary interventions (whole grain, low-gluten and refined grain), ranging up to 5% of initial body weight for 98% of the study participants. This is in line with the observation that individuals participating in randomized controlled trials tend to lose weight, independent of the intervention arm, if the study participants have measurements of body weight during the intervention^27^. Baseline body weight may also play a role in this regard.

Random forest models were trained across 50 shuffle-split cross-validated models for robust performance estimation. We found that only including information about the type of diet (whole grain-rich, low-gluten or refined grain diet) lead to a predictive performance of ROC-AUC: 0.62. Information on the type of diet is therefore only predictive of weight loss in some individuals. Despite a previously reported correlation between change in body weight and change in energy intake in the whole grain trial^25^, the total energy intake did not improve the predictive performance of weight loss together with age, sex and the type of diet (ROC-AUC: 0.57). As other biomarkers may predispose individuals towards a weight loss, we integrated information about host genetics, urine metabolome, physiological measures, postprandial response, whole shotgun gut microbiome sequencing and 16S rRNA amplicon sequencing data measured before the start of the dietary interventions. These were integrated together with diet in random forest models, where rigorous feature selection assisted with heterogenous data integration.

Features of the intestinal microbiome and urine metabolome were the most predictive of weight loss in combination with the type of diet, and boosted performance from a diet-only model from ROC-AUC 0.62 to 0.86–0.90. Several models of weight loss responders and non-responders were trained with different feature combinations, where we evaluated the importance of the features selected in the highest-ranking models (selected in minimum 15% of all models). For the 16S-based OTUs, the family *Ruminococcaceae* and genus *Streptococcus* were the most important features*. Ruminococcaceae* is one of the most abundant firmicutes families in the human gut and metabolizes plant material into short chain fatty acids (SCFA)^28^. *Ruminococcaceae* has been observed in higher abundance in obese Mexican women^29^, but also been associated with lower risk of obesity, cardiometabolic diseases^30^ and lower BMI^31^, and are found at lower abundance in Indian type 2 diabetes patients^32^. Decreased abundance of *Streptococcus* has been found in the development from glucose intolerance to type 2 diabetes^33^, and higher abundance in normal weight vs obese Mexican woman^29^. Some species of *Streptococcus* have been associated with weight loss and reduced fat accumulation in mice^34^.

The most important MGmapped gut microbiome species towards prediction included *F. prausnitzii*, *E. ramulus* and *R. faecis.* We pre-selected butyrate-producing species as these are associated to metabolic and intestinal health^35^, of these, the most important included *F. prausnitzii*, *E. ramulus* and *R. faecis. F. prausnitzii* has been shown to be associated with metabolic health in various studies due to anti-inflammatory properties from butyrate production. It has also been linked to obesity where the abundance is lower than in the healthy metabolic states^36^. *E. ramulus* has been associated with insulin resistance or dyslipidaemia in obese postmenopausal women^37^. In combination with LC–MS characterised urine metabolites, these microbiome features were most predictive and would be recommended for follow-up as potential weight loss predictive signals in future studies. For most of these species, there were no significant differences in the prevalence between responders and non-responders indicating non-linear combinations of features have been predictive of the weight loss response reflecting the nature of random forest models.

The implementation and use of an ensemble approach with heterogenous models, each requiring a different combination of input features, showed to be more resilient to missing data in the prediction of weight loss responders or non-responders, and had the highest performance of ROC-AUC: 0.86. In this regard, it was notable that omission of information of gut microbiome and urine metabolome features, resulted in a predictive performance of ROC-AUC: 0.72 using host genotype, gastrointestinal transit time and selected physiological features. Further, it allowed combining confident predictions per individual, thus using models that are most suited for that individual. We believe that such artificial intelligence (AI) frameworks can be useful as they integrate complex correlations across heterogenous data and facilitate discovery of signatures that potentially predispose to weight loss following a dietary intervention. AI frameworks may be developed to function as screening tools to assist in comprehensive strategies for weight management. For example, dietary interventions that are unlikely to benefit an individual may be deprioritised in favour of other weight loss strategies. Our AI models are able to identify 64% of the non-responders with 8 out of 10 correctly classified (NPV = 0.83), which is fairly promising.

## Limitations of study

While there is some agreement that a 5% to 10% weight loss goal is considered successful long term weight loss, consensus is limited on what constitutes significant short-term weight loss on an individual level^5,38^ and more importantly, normal body weight fluctuation is relatively unknown^39,40^. Thus, we recommend future studies of short-term weight loss to collect several longitudinal body weight measurements per individual in order to determine what can be deemed as significant personalised weight loss considering the daily fluctuations of an individual.

The study did not include information on exercise habits or the specific polysaccharide composition for starches and fibers, which are both known to have an impact on weight loss^41,42^. However, study participants were informed not to make life-style changes in the beginning of the clinical trials to avoid changes in exercise habits. Given day-to-day weight fluctuation, a single point cut-off definition of weight loss used in this study may insufficiently capture clinically significant weight loss for every individual. However, it was not possible to restrict the machine learning analysis to the responder extremes (e.g. by upper and lower quantiles) as there was too little data to run the models. The data used for modelling was obtained from clinical cross-over studies in 102 participants with two baseline time points per individual. Although deeply phenotyped, this is considered a limited number of individuals for effective data integration and machine learning. The urine metabolomics clearly improved performance when coupled with microbial species, but most of the useful features lacked annotation from the metabolomics pipelines. Finally, our robustness tests go some way in assessing performance as retraining models using randomly permutated prediction class labels and features selected by the models trained on the true prediction class labels resulted in a completely random ROC-AUC. Throughout the various evaluation setups, the best features prevailed, and it was good confirmation to see prior knowledge in microbial species – the butyrate producers – contributing substantially to the highest performing models. It was clear that diet alone had only limited predictive capability, whereas microbiome and metabolomic features substantially improved performance. Eventually, validation on independent cohorts would be meaningful to gain more confidence in the driving factors of individual weight loss.

## Methods

### Clinical studies design

The study protocol, randomisation, inclusion and exclusion criteria, and study products in the clinical studies that intervened with a whole grain-rich diet (https://clinicaltrials.gov, ID-no: NCT01731366) or a low-gluten diet (https://clinicaltrials.gov, ID-no: NCT01719913) are described previously^22^. Both studies were performed in accordance with relevant regulations and written informed consent was obtained from all participants. The studies were approved by the Ethical Committee of the Capital Region of Denmark in accordance with the Helsinki Declaration (H-2-2012-065) and the Data Protection Agency (2007-54-0269). The two studies consisted of two 8 week-intervention periods separated by a 6 weeks washout period. The whole grain-rich intervention aimed at an intake of $\ge$ 75 g whole grain per day and the target for the low-gluten diet was $<$ 2 g/day of gluten. Both studies used the same refined grain diet as control, which was designed to contain $<$ 10 g whole grain/day and $>$ 20 g gluten/day. The studies recruited a total of 120 healthy Danish men and women (60 subjects for each study), who were generally healthy, but should be overweight (defined by BMI or waist circumference) and have two other risk markers for the metabolic syndrome (high blood pressure, plasma glucose, or triglyceride or low HDL-cholesterol).

For detailed experimental produces and analyses of the collected data, we refer to the “Methods” sections in previously published papers^25,26^. In brief, the study participants attended an examination before and after each intervention periods. The examinations were scheduled in the morning, where study participants were instructed to be fasted ≥ 10 h overnight, to avoid tooth brushing and smoking, and to abstain from alcohol and exercise ≥ 24 h. The participants had a physical examination and fasting blood and urine samples, as well as faecal samples were collected. The physical examination consisted of blood pressure measurements and anthropometrics including measurements of body weight, sagittal abdominal diameter, waist circumference, and body composition by bioelectrical impedance analysis.

The blood samples were analysed for various biomarkers of glucose and lipid metabolism, markers of inflammation and liver health, such as glucose, insulin, cholesterol, triglyceride, IL-6, CRP and alanine aminotransferase and aspartate aminotransferase. Urine samples, and faecal samples were collected. Gut permeability was assessed by lactulose and mannitol secretion in the urine, while transit time was measured by X-ray after ingestion of 24 radiopaque markers^25,26^. The subjects were also asked to fill out a self-reported questionnaire of overall well-being and gastrointestinal symptoms using a visual analogue scale (VAS) and to keep a study diary to monitor dietary compliance. In addition, the study participants consumed a standardized breakfast^22^ to assess their post-prandial response. The fasting blood sample was used as time 0 and samples were obtained again 30, 60, 120 and 180 min after the meal. The samples from all five time points in the time series were analysed with focus on glucose regulation and appetite hormones. Breath hydrogen (H_2_) was measured twice at fasting and then seven times with 30-min intervals after the meal, giving a total of eight time points.

### Weight loss responders and non-responders outcome

We focused on identifying weight loss responders and non-responders. We considered body weight (kg) where individuals were grouped for classification by assessing the relative individual change between visit 1 and 2, and between visit 3 and 4 independent of the dietary study arm. The relative change is calculated as ${\Delta }_{w}=\frac{{w}_{after}-{w}_{before}}{{w}_{before}}$, where *w* is the body weight. The responders and non-responders to diet were defined by individuals losing weight or not during the dietary intervention periods, i.e. ${\Delta }_{weight}\ge 0$ are the non-responders, and ${\Delta }_{weight}<0$ are the responders.

In this study, we investigated which data types were predictive of weight loss using baseline biomarkers and what features were most important by the machine learning models. In order to compare this across models, we thus only used data types available in all individuals (N = 130). Secondly, we made an ensemble of selected models using only their confident predictions. In these ensembles, models are included in the assessment only when the model is confident of its assertion in order to distinguish the weight loss responders and non-responders with high certainty.

### Gut microbiome

Faecal samples were sequenced by 16S rRNA amplicon sequencing and by shotgun sequencing. Taxonomies were annotated from the 16S data using QIIME2 tool^43^ with default quality filtering parameters. The annotation process was completed in three steps: pre-processing, selection of representative sequences and assigning taxonomies. OTU clusters are generated using the Deblur sub-OTU method. As default, all the OTU clusters with abundances less than 2 or 0.005% were removed, then assigned to taxonomies using the SILVA 128 reference database^44^. 10,093 unique OTU clusters passed the quality control. The unique OTU clusters were assigned to different levels of taxonomy from kingdom to species. Relative taxonomy abundances were calculated as the ratio between the obtained taxonomy abundances in a sample and the total taxonomy abundances of the sample.

Shotgun metagenomic sequencing (Illumina paired 2 × 150 nt) was used to generate metagenomic species (MGS) by mapping reads to human gut microbiome reference genes from the integrated gene catalogue (IGC) as previously described^25,26^. In addition, shotgun sequenced Illumina paired-end reads were mapped using MGmapper version 2.7 with five reference databases: Human Microbiome, Meta Hit Assembly, Bacteria, Bacteria Draft, Human and Fungi. Only taxonomical species annotated by the Human microbiome reads, Bacteria reads and Bacteria Draft reads were used in the data analyses. The Meta Hit Assembly was skipped, after we found no butyrate-producing species in the catalog. The Human and Fungi catalogues were not used due to too few mapped reads. MGmapper uses similarity-based mapping with the BWA-mem algorithm to find taxonomies in a specified database with pre- and post-processing of the raw reads to lower the number of false positives in the taxonomy annotation^45^. The mapping was compiled as species relative abundances, which are calculated as $S\_abundance=100\cdot \frac{ReadCount}{Size \cdot 2}$ for the paired-end reads, where the *Size* is the length of the reference sequence in base pairs.

### Urine metabolomics

Urine samples were analysed by gas chromatography-mass spectrometry (GC–MS) and liquid chromatography–mass spectrometry (LC–MS) (in both positive and negative ionization mode) as previously reported^25,46^. Metabolites measured by LC–MS were putatively annotated using the metabolites’ mass, retention time and mode by searching features of interest against the Human Metabolome Database^47^ and Metlin Database^48^ and annotated at level 3–4 as described by the Metabolomics Standard Initiative^49^.

### Genotype

DNA was extracted from human blood leucocyte nuclei and were genotyped by Infinium CoreExome-24 BeadChip (Illumina, San Diego, CA). Genotypes were called from Genome studio using the human genome assembly GRCh37 as calling reference. 117 study participants were genotyped for 547,644 single nucleotide polymorphisms (SNPs) after updating genome to build 37. Quality control and genome-wide association study (GWAS) was performed using PLINK1.9^50^ for sample and SNP call-rates (98%), sex check, excess heterozygosity and homozygosity, inbreeding, pedigree (relatedness), removal of non-European ancestry, Hardy-Weinberg Equilibrium (HWE, 0.005) and minor allele frequency (MAF, 1%) resulting in 105 samples and 272,588 SNPs.

### Genome-wide association study and genetic risk scores

To reduce the feature input space for the 272,588 genetic variants, we utilized three different approaches to prioritise SNPs for modelling of weight loss. First, we performed a literature study on genes involved in metabolic pathways, inflammation and gut microbiome composition (Supplementary Material 2a). The SNPs were annotated to these genes using Ensembl variant effect predictor^51^ (human build GRch37) with default parameters (Upstream/downstream gene distance of 5 kb) resulting in 703 unique SNPs available on the platform after QC within the set gene distance boundaries. The selected SNPs were linkage-disequilibrium pruned using PLINK1.9 –indep to leave only independent SNPs based on the variance inflation factor, VIF (parameters: window size 50 kb, step size 5 and VIF threshold 1). This resulted in 56 SNPs for modelling. Furthermore, SNPs were binary encoded according to the presence of major and minor alleles.

In addition, to test the hypothesis that genetic risk variants for obesity, overweight, body weight and sagittal abdominal diameter are important in predisposing individuals to weight loss and maximize the power for genetic information, we developed five weighted genetic risk scores (GRS) with 4–10 SNPs in each based on a total of 32 SNPs (Supplementary Material 2d). The GRSs were calculated as the sum of the number of minor alleles multiplied by the effect size of the SNPs. Two GRS were based on a GWAS using data from the whole grain trial. A linear regression model was applied to perform GWAS on the weight changes and sagittal abdominal changes ${\Delta }_{whole\,grain{-}rich \,diet}-{\Delta }_{refined\, grain\, diet}$. This phenotype assumes the changes are only caused by the dietary interventions to capture genetic predisposition to changes. If the phenotype for GWAS did not follow a normal distribution, it was converted into a z-score prior to association analysis by linear regression. Age, sex and randomisation order of the intervention treatments were included as co-variates.

Three other GRS’s were based on a literature study on SNPs involved in metabolic health using the NHGRI-EBI GWAS catalogue^52^ with the search keyword “obesity”. A study was considered for calculation of a GRS if more than one SNP was present in our data and the particular SNP had a p-value $<{10}^{-4}$.

### Postprandial response to standardized meal test

The postprandial response to a standardized test meal was measured using four biochemical markers from blood samples being; free fatty acids, GLP-2, glucose and insulin and in breath hydrogen. After a 10-h fasting period, the first blood was sampled and samples were obtained again at 30, 60, 120 and 180 min after a standardized breakfast, for a total of five time points in the time series. Breath hydrogen (H_2_) was measured twice at fasting and averaged and then seven times every 30 min following the meal, giving a total of eight time points. Typically, the postprandial response is represented by area under the postprandial curve, which does however not capture information about temporal variation in data. We thus modelled the dynamics of the postprandial response by three new feature representations of volatility. First, the fluctuation was measured as movement of the values between time points by differencing the time points consecutively and summing up their absolute differences using following formula:

$$fluc1=\frac{{\sum }_{i=2}^{len(x)}abs({x}_{i}-{x}_{i-1})}{len(x)}$$

For this calculation, we used the normalized time series and we called the outcome measure fluc*1*. Secondly, we plotted the non-normalized time series of the measures for each patient and analysed the fluctuation by its graph. In the analysis of the graph, interpolation of the series was used to add more time points. If the series had one missing value the interpolation would be used to replace the missing value; if there were more than one missing value, the representations would be set as 0. The interpolation function interp1d from the SciPy (version 1.2.1) Python package was used with 100 interpolated points and a spline interpolation (kind = “cubic”). Each graph was then divided into a grid of size 10 × 10 and 50 × 50 all with the same axis scale based on the maximum and minimum value in the data of the postprandial marker. From the grid division the 100 interpolated time points were interpreted into an image vector consisting of 1 s and 0 s for squares with and without points, respectively. This was done vertically with each column being interpreted from the lower boundary to the upper and concatenated into one vector. The image vector was summed, and we called the outcome measure *fluc2*. The third measure we got from filtering the sum, thus it would only allow for addition between squares with two or more consecutive 1 s. This measure we called *fluc3*.

### Data integration and machine learning strategies

Data collected from before the two interventions for all participants was used to explore predictive biomarkers of weight loss in machine learning models. The type of diet (whole grain-rich, low-gluten or refined grain diet) was included in all models to differentiate between the two baselines for the same participant. We only included study completers for modelling. Random forest classifiers modelled the samples using 50 shuffle-split fivefold cross-validation stratified by target classes. The models were made in Python (version 3.7.1) using the package Scikit-learn (version 0.20.1) for machine learning methods, especially the class RandomForestClassifier(), which was used for modelling. The number of decision trees in the forest was fixed at n_estimators = 50 and model initialization was fixed at random_state = 42 for reproducibility. The number of features considered at each split was set as max_features = None, meaning that the random forest could use all features for a split. To limit the forest growing into overfit the minimum decrease in impurity required to make a split was set to min_impurity_decrease = 0.01, meaning that there had to be at least 1% decrease in impurity^53^.

### Model and feature importance evaluation

The predictive performance of the machine learning models was assessed as the area under the Receiver Operating Characteristic (ROC) curve (ROC-AUC). The ROC is a graph showing the true positive rate (TPR) against the false positive rate (FPR), when the threshold is varied for labelling a data point as either positive or negative in a binary classifier. In addition, we reported sensitivity, specificity, and Matthews Correlation Coefficient (MCC). Permutation tests were applied in order to assess if the models would perform more randomly when a shuffled set of target labels were used. These tests were performed by random shuffling of the class labels 50 times followed by training and testing on the shuffled target labels, as well as comparing the performance ‘random’ models to the models trained and tested on the true target labels. The shuffle was applied only 50 times, due to the limited sample size, as the amount of very different sets that can be generated at random is lower. Three permutation setups are reported; (i) The classifier was re-trained using permuted class labels and the selected features by the models trained on the true target. (ii) A classifier with permuted class labels was allowed to train and optimize its feature selection on the permuted prediction labels, meaning that it tries to find the best possible fit for the randomly shuffled data labels. Thereby, a high performance is potentially caused by the fact that the shuffled model learns a pattern in the noise—a risk in any data-driven approach. iii) The model trained on the true class label and test is stored and performance is evaluated on the shuffled target labels.

The importance of features in the random forest models was evaluated using the Gini index^54^. The Gini index feature importance is part of the random forest algorithm, which evaluated how many times a given feature was involved in a node split. This will be shown as the Mean Decrease in Impurity (MDI), i.e. how much a variable on average contributes to the decrease in node impurity. The averaged importance of a feature, if all are assigned similar importance, should be $imp=\frac{1}{M}$, where M is the number of features in a model, since the feature importance sum to 1.

### Feature selection

For data types and combinations with higher dimensionality, additional means of feature selection were applied, which used both prior knowledge and data-driven approaches to lower the feature space when optimizing the models to avoid overfitting. Features for the prior knowledge approach were selected up-front, while the features selected from the data-driven approach were assessed through the 50 fivefold shuffle-split cross-validation.

#### Prior knowledge feature selection

We prioritized features in the microbiome data from 16S taxonomies, MGmapped species, MGS’ and in the genotype data. The prioritization and representation of genotype data is described previously in the “Methods” section “Genome-wide association study and genetic risk scores”. For the microbiome data from 16S taxonomies, we assessed the prevalence and variance between visits and used this to select the top 10 and top 250 16S-based OTUs for the machine learning models. 16S-based OTUs present in at least 5 people were considered. The prevalence and variance were used as a proxy for selecting taxa with high information.

For the MGmapped gut microbiome species, we prioritized 17 butyrate-producing species identified in previous studies and which were available in the MGmapper datasets mapped against the catalogues Bacteria, Bacteria draft and Human Microbiome (Supplementary Material 2b). Of the 17 unique butyrate-producing species, the Bacteria catalogue had nine microbial species, the Bacteria draft catalogue had 11 microbial species and the Human Microbiome catalogue had 10 microbial species. *Anaerostipes caccae *was removed from analysis, since it was found to have an abundance of 0 in all study participants at the two baselines in the Bacteria draft catalog.

For the MGS’, we prioritized the top altered species from the whole grain and gluten studies^25,26^. For the gluten study, 14 MGS’ were significantly altered when comparing the changes to abundance on refined grain diet and low-gluten diet. In the whole grain study, no MGS’ were significantly altered when comparing the changes to abundance on refined grain diet and whole grain-rich diet. The top 14 most altered MGS’ were therefore selected from the whole grain study as well. This resulted in 28 pre-selected MGS’.

#### Data-driven feature selection

We did an exhaustive feature selection on the metabolome data with all possible subset pairs or triplets of metabolites in order to assess if any subset could improve predictive power. For that, the random forests were run with each subset and ROC-AUC performances compared.

Forward selection was also applied to many combinations of different data sets. This selection was performed by adding one feature at a time and then check which feature combinations increased ROC-AUC in the cross-validation. When multiple equally good features were found, all are first added to see if this performs better. If the new model is not better, one of the equally good features is randomly selected. However, the other features are still in the pool and can be selected at a later iteration. The worst performing features were gradually removed at each iteration in order to save computation time. This continued until performance no longer increased, and the optimal model was saved. The feature selection has been made with a set of parameters which include a list of features to select from (selects), the maximum number of features to select (max_features), the initial fraction of features to remove at each iteration (frac) and the step size of removing features (step), which is updated after a feature is added. The list of features to select from depended on which data sets where included, and the features not shown in this list were added before the first iteration. The maximum number of features to select was set to max_features = 8 or unlimited (giving ~ 5–15 selected features). The initial fraction for removing the lowest performing features was frac = 0.4, meaning that the 40% worst performing features are removed in the first iteration. The step size was set to step = 0.1, thereby the number of removed features was 10% less at each iteration. Once the fraction became less than 0.1, the step size is changed to 0.01 automatically, and when this fraction results in 0, a single feature will hence forth be removed at each iteration.

### Statistical analyses

Statistical testing of distributions (responder and non-responders and permutation analysis of the ROC-AUC distributions) were assessed by a two sampled unpaired t-test if data followed a Gaussian distribution or Mann Whitney test if non-Gaussian distribution. A p-value < 0.05 was considered significant.

### Personalised artificial intelligence ensembles

The data types were combined into different sets in order to determine how they might capture different aspects of the data, which were reported by an ensemble model. The ensemble model is built based on the prediction scores from multiple models (50 shuffle-split fivefold cross-validation models). We created different ensemble models by different confidence predictive thresholds {s = [≤ 0.30, ≥ 0.70], [≤ 0.25, ≥ 0.75], [≤ 0.20, ≥ 0.80]} and by four scoring methods for which each sample is evaluated across all states based on prediction scores. This yields a final set of scores or predictions per sample, for which the ensemble performance can be evaluated. The scoring methods are:

1. *Mean of scores:* The mean of prediction scores.
2. *Majority voting:* The prediction score for each model is rounded to either 0 or 1, for the classes non-responder and responder, respectively. The predicted class chosen by most models will be the ensemble prediction.
3. *Confident mean of scores:* The mean of the prediction scores that is considered “confident”, based on a set threshold. If this was set to e.g. 0.7, then all samples with prediction score equal to/below 1–0.7 = 0.3 or equal to/above 0.7 would be considered confident scores to be included in the mean score. If a sample has no confident scores, it is excluded from the performance calculation for the ensemble.
4. *Majority voting on confident scores:* A mixture of 2) and 3). The predictions that are considered “confident” based on a threshold are rounded to either 0 or 1 for the non-responder and responder classes. The predicted class is the one chosen by most models in the ensemble.

### Prediction of individuals at high confidence of weight changes

The predictions made with these models are the probabilities of a sample belonging to either class 0 (non-responders) or class 1 (responders). The class probabilities for each tree are estimated as the fraction of samples belonging to the same class in each leaf of the tree. As the random forest consists of multiple decision trees, the class probabilities are predicted as a mean of the predicted class probabilities for each tree in the forest. By thresholding the prediction probabilities, we can at a given probability define the number of participants that we are sure will or will not experience weight loss. To evaluate this, we reported the sensitivity, specificity, positive predictive value (PPV) and negative predictive value (NPV).

## Supplementary information

Supplementary Information 1.

Supplementary Information 2.

Supplementary Information 3.

## Data Availability

The raw Illumina read data for all whole grain study samples have been deposited to the Short Read Archive database [https://www.ncbi.nlm.nih.gov/sra] with the accession number PRJNA395744. The raw Illumina read data for all gluten study samples have been deposited to the Short Read Archive database [https://www.ncbi.nlm.nih.gov/sra] with the accession number PRJNA491335. Other datasets generated during and/or analysed during the current study are available from the corresponding author on reasonable request.

## Code availability

Code for machine learning setup including random forest and ensemble models can be obtained from; https://github.com/disease-data-intelligence/3G_weight_loss_prediction.
