# Supplementary material for: Data integration for prediction of weight loss in randomized controlled dietary trials
Source: Sci Rep. 2020 Nov 18;10:20103. doi: 10.1038/s41598-020-76097-z (PMC7674420; doi:10.1038/s41598-020-76097-z)
Supplement: Supplementary file 1 — Supplementary Information 1. [file 41598_2020_76097_MOESM1_ESM.pdf]

Supplementary Material

Data integration for prediction of weight loss in randomized controlled dietary trials

Rikke Linnemann Nielsen[1,2*], Marianne Helenius[1*], Sara L. Garcia[1], Henrik M. Roager[3,4], Derya Aytan-Aktug[1,4,] Lea Benedicte Skov Hansen[1], Mads Vendelbo Lind[3], Josef K.Vogt[6], Marlene Danner Dalgaard[1], Martin I. Bahl[4], Cecilia Bang Jensen[1] , Rasa Muktupavela[1], Christina Warinner[5], Vincent Aaskov[6], Rikke Gøbel[6], Mette Kristensen[3], Hanne Frøkiær[7], Morten H. Sparholt[8], Anders F. Christensen[8], Henrik Vestergaard[6,9], Torben Hansen[6], Karsten Kristiansen[10], Susanne Brix[11], Thomas Nordahl Petersen[4], Lotte Lauritzen[3**], Tine Rask Licht[4**], Oluf Pedersen[6**], Ramneek Gupta[1,12**].

* These authors contributed equally

** Corresponding authors

E-mail, corresponding author Ramneek Gupta: ramg@dtu.dk

Affiliation

1. Department of Health Technology, Technical University of Denmark, DK-2800, Kgs. Lyngby, Denmark.
2. Sino-Danish Center for Education and Research, University of Chinese Academy of Sciences, Beijing, China.
3. Department of Nutrition, Exercise and Sports, University of Copenhagen, Denmark.
4. National Food Institute, Technical University of Denmark, Denmark.
5. Department of Anthropology, Harvard University, Cambridge, USA 02138.

6. The Novo Nordisk Foundation Center for Basic Metabolic Research, Faculty of Health and Medical Sciences, University of Copenhagen, DK-2200, Copenhagen, Denmark.
7. Institute for Veterinary and Animal Sciences, University of Copenhagen, Frederiksberg, Denmark.
8. Department of Radiology, Bispebjerg Hospital, Copenhagen, Denmark.
9. Department of Medicine, Bornholms Hospital, Rønne, Denmark
10. Laboratory of Genomics and Molecular Biomedicine, Department of Biology, University of Copenhagen, DK-2100, Copenhagen, Denmark.
11. Department of Biotechnology and Biomedicine, Technical University of Denmark, Denmark.
12. Novo Nordisk Research Centre Oxford, Oxford, OX3 7FZ, United Kingdom.

Supplementary Material 1: Flowchart of machine learning framework and data integration strategy

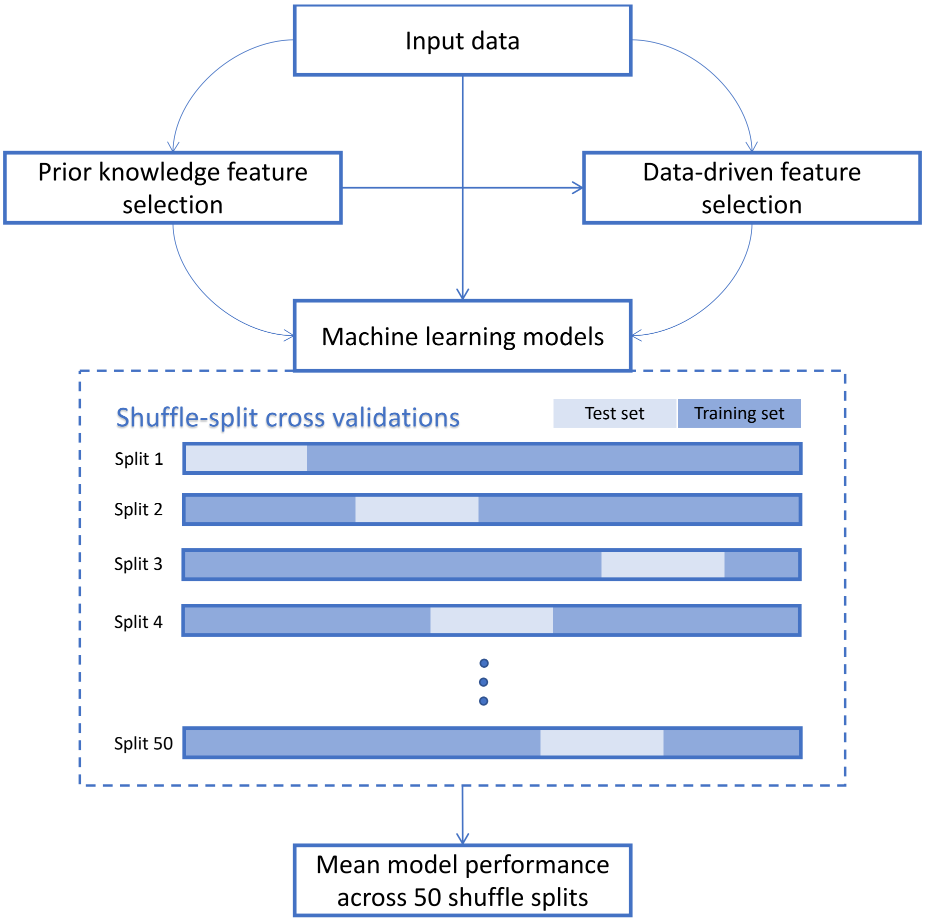

Figure S.1: **Feature selection and machine learning setup.** The feature selections were performed either using prior knowledge approaches or by a data-driven approach. For the prior knowledge selection approaches, features of interest were selected upfront based on e.g. literature and then used to train and test models through 50 five-fold shuffle-split cross-validations. The data-driven approach was done by iteratively evaluating different sets of features in the cross-validation setup and store the found best combination. We ensured that the model initialization was constant but varied the cross-validation splits by applying a 50 five-fold shuffle-split cross-validation setup as illustrated in this figure.

Supplementary Material 2: Genes from literature pathways, butyrate-producing species from literature study, top metagenomic species and SNPs used in genetic risk scores

Table S.2a): **Biological aspect, pathways, genes and literature references used to select 703 SNPs for the LitPath and LitPathLD dataset**. The SNPs for modelling were found by annotation to SNPs to genes by Variant Effect Predictor (VEP build 37).

Biological aspect	Pathways	Genes	Reference
B12 and folate pathways	B12 and folate pathway	ABCD4, CD320, CLYBL, CUBN, FOLR3, FUT2, FUT6, MMAA, MMACHC, MTHFR, MUT, TCN1 and TCN2	Grarup, Niels; Sulem, Patrick; Sandholt, Camilla H; et al. Genetic architecture of vitamin B12 and folate levels uncovered applying deeply sequenced large datasets. P L O S Genetics — 2013, Volume 9, Issue 6
Cancer, fibrin/collagen formation and anti-xenobiotic effects	Various that affect cancer, fibrin and collagen formation and anti-xenobiotic effects.	ACTA2, ACTG2, AKR1B10, CAPN13, CAPZA2, CES1, COL6A1, CYP1A1, CYP2D6, CYP3A7, CYP4A22, CYP3A66, cytochrome P450, DHDH, FXYD3, GOLPH2, GSN, GSTM4, GSTM5, KRT18, MT1E, MT1F, NAD, PTTG1, p24, RETNLB, SULT2A1, S100P, UGT2B10, UGT2B11 and ZBTB16	Sestak K, Conroy L, Aye PP, Mehra S, Doxiadis GG, et al. (2011) Improved Xenobiotic Metabolism and Reduced Susceptibility to Cancer in Gluten-sensitive Macaques upon Introduction of a Gluten-Free Diet. PLoS ONE 6(4):e18648
Fatty acid metabolism and cytokine signalling	PPAR signalling pathway (activation of Fatty acid degradation in liver) and cytokine signalling (FAS/TNFRSF6, MCP-1, TNFalpha and IL-6)	CPT1, FAS, IL-6, L-FABP, MCP-1, PPAR-a, SCD1, TNF-α and UCP2	Park, Mi-Young; Jang, Hwan-Hee; Kim, Jung Bong; et al. Hog millet (Panicum miliaceum L.)-supplemented diet ameliorates hyperlipidemia and hepatic lipid accumulation in C57BL/6J-ob/ob mice. NUTRITION RESEARCH AND PRACTICE — 2011, Volume 5, Issue 6, pp. 511-519
Gut microbiota modulation	ABO blood group	FUT2	Rausch et al., Colonic mucosa-associated microbiota is influenced by an interaction of Crohn disease and FUT2 (Secretor) genotype, PNAS, vol. 108, no. 47, 19030–19035 (2011), -Mäkivuokko et al., Association between the ABO blood group and the human intestinal microbiota composition, BMC microbiology, 12:94, (2012), Wacklin et al., Secretor Genotype (FUT2 gene) is Strongly Associated with the Composition of Bifidobacteria in the Human Intestine, Plos One, vol 6, 5, e20113 (2011), and more
	G-protein coupled receptor	FFAR2, FFAR3, GPR109a, GPR41 and GPR43	Aw W and Fukuda S. Toward the comprehensive understanding of the gut ecosystem via metabolomics-based integrated omics approach. Semin Immunopathol 2014. DOI 10.1007/s00281-014-0456-2
	HDL complex	APOA1	Zhang, C. et al. Interactions between gut microbiota, host genetics and diet relevant to development of metabolic syndromes in mice. ISME J. 4, 232–241 (2010).
	IFN signalling	IFN-α, IFN-ß, IFN-γ, IFNG, IFR9 and STAT4	Thompson, C. L., Hofer, M. J., Campbell, I. L. & Holmes, A. J. Community dynamics in the mouse gut microbiota: a possible role for IRF9-regulated genes in

			community homeostasis. PLoS ONE 5, e10335 (2010)
	Tol-Like receptor signalling	TLR2 and TLR5	Albert, E. J., Sommerfeld, K., Gophna, S., Marshall,<br>J. S. & Gophna, U. The gut microbiota of toll-like receptor 2-deficient mice exhibits lineage-specific modifications. Environ. Microbiol. Rep. 1, 65–70 (2009).<br><br>Vijay-Kumar, M. et al. Metabolic syndrome and altered gut microbiota in mice lacking Toll-like receptor 5. Science 328, 228–231 (2010).
	Various	ANG, IFN, IGF1, IL-1B, IL-17B, IL-23, IRAK2, JUN, OXT, POMC, PYY, STAT4, TLR3, TLR9 and UCN	van Baarlen, Peter, ; Troost, Freddy; van der Meer, Cindy; et al. Human mucosal in vivo transcriptome responses to three lactobacilli indicate how probiotics may modulate human cellular pathways. PROCEEDINGS OF THE NATIONAL ACADEMY OF SCIENCES OF THE UNITED STATES OF AMERICA — 2011, Volume 108, Issue Suppl. 1, 1, pp. 4562-4569
Immune response	Cytokine signaling, G protein signaling, T cell activation	CCR3, IL12A, IL18RAP, RGS1, SH2B3 and TAGAP	K. A. Hunt, A. Zhernakova, G. Turner et al., “Newly<br>identified genetic risk variants for celiac disease related to the<br>immune response,” Nature Genetics, vol. 40, no. 4, pp. 395–<br>402, 2008.
	Production of PAI-1, NADPHox, iNOS, TLR4, THF-a, (gut permability)	iNOS, NADPHox, PAI-1, THF-a and TLR4	Cani, P. D., ; Possemiers, S.; Van de Wiele, T.; et al. Changes<br>in gut microbiota control inflammation in obese mice through a<br>mechanism involving GLP-2-driven improvement of gut permeability. GUT — 2009, Volume 58, Issue 8, pp. 1091-1103.
Immune response, lipogenic/adipogenic regulation, and cell proliferation and differentiation	Transcription factors in immune, lipid and cell regulation	NF-kB, NEMO, akt, PPARg and SREBP1	Radonjic, Marijana; de Haan, Jorn R.; van Erk, Marjan J.; et al. Genome-Wide mRNA Expression Analysis of Hepatic Adaptation to High-Fat Diets<br>Reveals Switch from an Inflammatory to Steatotic Transcriptional Program. PLOS ONE — 2009, Volume 4, Issue 8, pp. -
Inflammation	Cytokine signalling (IL-6 response by CRP and TNF-R2)	CRP and TNF-R2	Mantzoros, Christos,Franz, Mary Van Dam, Rob M et al. Wholeo-grain, bran, and cereal fiber intakes and markers of systemic inflammation in diabetic women. Diabetes Care 29:207–211, 2006.
Insulin secretion	Wnt signalling pathway	TCF7L2	Fisher E, Boeing H, Fritsche A, et al. Whole-grain consumption and transcription factor-7-like 2 (TCF7L2) rs7903146: gene–diet interaction in modulating type 2 diabetes risk. Br J Nutr 2009; 101:478–481.
Potential gluten degrading bacteria	NOD-like receptor signalling pathway	MEFV	Khachatryan, Z. A. et al. Predominant role of host genetics in controlling the composition of gut microbiota. PLoS ONE 3, e3064 (2008)
Signal transduction, metabolism (other than energy metabolism), cell cycle control, transcription and	Various that influence Signal transduction, metabolism (other than energy metabolism), cell cycle control, transcription and translation, control of	ALDH7A1, ATP6V0B, BAP1, CDNA, CLY6G5B, CTNNB1, DGKD, DHCR24, DIO2, D123, EFNB2, EGFR, FLJ43113, FTCD, HIBADH, MRPL4, MTCP1, PKIA, PIK3R1, RIT1,	K. Juuti-Uusitalo, M. Mäki, H. Kainulainen, J. Isola and K. Kaukinen. Gluten affects epithelial differentiation-associated genes in small intestinal mucosa of coeliac patients. 2007 British

translation, control of cellualar organization and transport facilitation	cellualar organization and transport facilitation	RLF, RNTRE, ROK1, SFA1, TM4SF1, WAVE1 and ZNF161	Society for Immunology, Clinical and Experimental Immunology, 150: 294–305
Transit time	Incretin response	GCG	Anita Wichmann, Ava Allahyar, Thomas U. Greiner, Hubert Plovier, Gunnel Östergren Lundén, Thomas Larsson, Daniel J. Drucker, Nathalie M. Delzenne, Patrice D. Cani, Fredrik Bäckhed, Microbial Modulation of Energy Availability in the Colon Regulates Intestinal Transit, Cell Host & Microbe, Volume 14, Issue 5, 13 November 2013, Pages 582-590
Genes related weight, obesity or metabolic syndromes		FAIM2, FBXO22, FTO, NRG4, PAK7, PLCB1, SEC16B, SH2B1 and UBE2Q2	De Giorgio MR, Yoshioka M, St-amand J: Feeding induced changes in the hypothalamic transcriptome. *Clinica Chimica Acta* 2009, 406:103–107.<br><br>Frazier-Wood, A. C. & Wang, Z. Genetics of Obesity. in *Metabolic Syndrome* 123–140 (Springer International Publishing, 2016). doi:10.1007/978-3-319-11251-0_10<br><br>Mehta NK, Mehta KD: Protein kinase C-beta: an emerging connection between nutrient excess and obesity. *Biochimica et Biophysica Acta (BBA) - Molecular and Cell Biology of Lipids* 2014, 1841:1491–1497<br><br>Park, S. H., Lee, J. Y. & Kim, S. A methodology for multivariate phenotype-based genome-wide association studies to mine pleiotropic genes. *BMC Syst. Biol.* **5**, S13 (2011).<br><br>Wang G, Zhao X, Meng Z, Kern M, Dietrich A, Chen Z, Cozacov Z, Zhou D, Okunade AL, Su X, Li S, Blüher M, Lin JD: The brown fat – enriched secreted factor Nrg4 preserves metabolic homeostasis through attenuation of hepatic lipogenesis. *Nature medicine* 2014, 20:1436–1443.

Table S.2b): **Butyrate-producing species selected by literature study**, and the MGmapper catalogue they are present in. Some species were found in more of the references listed, but only the first it was found in is listed.

Species	MGmapper catalogue	Reference
Anaerostipes caccae	Bacteria draft and Human Microbiome	Petra Louis, Harry J. Flint, "Diversity, metabolism and microbial ecology of butyrate-producing bacteria from the human large intestine", *FEMS Microbiology Letters*, Volume 294, Issue 1, May 2009, Pages 1–8, doi: 10.1111/j.1574-6968.2009.01514.x
Anaerotruncus colihominis	Bacteria draft and Human Microbiome	
Butyrivibrio fibrisolvens	Bacteria and Bacteria draft	
Eubacterium hallii	Bacteria draft	
Eubacterium ramulus	Bacteria draft	
Faecalibacterium prausnitzii	Bacteria, Bacteria draft and Human Microbiome	
Roseburia faecis	Bacteria draft	

Roseburia hominis	Bacteria	
Roseburia intestinalis	Bacteria, Bacteria draft and Human Microbiome	
Roseburia inulinivorans	Bacteria draft and Human Microbiome	
Subdoligranulum variabile	Bacteria draft and Human Microbiome	
Anaerostipes hadrus	Bacteria and Human Micrbiome	Sato, T., Kusuhara, S., Yokoi, W., Ito, M. & Miyazaki, K. Prebiotic potential of L-sorbose and xylitol in promoting the growth and metabolic activity of specific butyrate-producing bacteria in human fecal culture. *FEMS Microbiol. Ecol.* **93**, fiw227 (2017). doi: 10.1093/femsec/fiw227
Bacteroides uniformis	Bacteria draft and Human Microbiome	K. Takahashi *et al.*, "Reduced Abundance of Butyrate-Producing Bacteria Species in the Fecal Microbial Community in Crohn's Disease," *DIG*, vol. 93, no. 1, pp. 59–65, 2016. doi: 10.1159/000441768
Clostridium butyricum	Bacteria	G. Cai, B. Jin, C. Saint, and P. Monis, "Genetic manipulation of butyrate formation pathways in Clostridium butyricum," *J. Biotechnol.*, vol. 155, no. 3, pp. 269–274, Sep. 2011. doi: 10.1016/j.jbiotec.2011.07.004
Clostridium kluyveri	Bacteria	H. Seedorf *et al.*, "The genome of Clostridium kluyveri, a strict anaerobe with unique metabolic features," *PNAS*, vol. 105, no. 6, pp. 2128–2133, Feb. 2008. doi: 10.1073/pnas.0711093105
Eubacterium limosum	Bacteria	S. Park *et al.*, "Acetate-assisted increase of butyrate production by Eubacterium limosum KIST612 during carbon monoxide fermentation," *Bioresour. Technol.*, vol. 245, no. Pt A, pp. 560–566, Dec. 2017. doi: 10.1016/j.biortech.2017.08.132
Fusobacterium nucleatum	Bacteria and Human Microbiome	M. Vital, A. C. Howe, and J. M. Tiedje, "Revealing the Bacterial Butyrate Synthesis Pathways by Analyzing (Meta)genomic Data," *mBio*, vol. 5, no. 2, pp. e00889-14, May 2014. doi: 10.1128/mBio.00889-14

Table S.2c): **Top 14 most altered metagenomic species from the whole grain and gluten studies[25,26].**

**Metagenomic species**	**Taxonomic annotation**	**Adjusted (FDR) p-value**	**Reference**
MGS:igc210	Lachnospiraceae (family)	5.42E-09	Skov, L. B. et al. A low-gluten diet induces changes in the intestinal microbiome of healthy Danish adults. 28, (2019).<br><br>Supplementary Data 2.
MGS:igc939	*Bifidobacterium angulatum*	2.98E-07	
MGS:igc413	*Bifidobacterium longum*	1.73E-06	
MGS:igc529	*Bifidobacterium adolescentis*	8.38E-06	
MGS:igc356	*Bifidobacterium pseudocatenulatum*	1.10E-04	
MGS:igc121	Lachnospiraceae (family)	1.07E-03	
MGS:igc846	*Dorea* (genus)	2.13E-03	
MGS:igc221	Unclassified	2.67E-03	

MGS:igc491	*Dorea longicatena*	7.02E-03	
MGS:igc47	*Blautia wexlerae*	7.34E-03	
MGS:igc1021	Unclassified	9.77E-03	
MGS:igc492	Clostridiales (order)	9.80E-03	
MGS:igc78	*[Eubacterium] hallii*	1.72E-02	
MGS:igc169	*Anaerostipes hadrus*	4.94E-02	
MGS:igc654	Clostridiales (order)	0.16	Roager, H. M. et al. Whole grain-rich diet reduces body weight and systemic low-grade inflammation without inducing major changes of the gut microbiome: a randomised cross-over trial. Gut 68, 83–93 (2019).<br><br>Online Supplementary Material 2, Table S7.
MGS:igc102	*Erysipelatoclostridium ramosum*	0.16	
MGS:igc460	Clostridiales (order)	0.16	
MGS:igc633	Clostridiales (order)	0.16	
MGS:igc139	*Ruminococcus* (genus)	0.18	
MGS:igc291	*Faecalibacterium prausnitzii*	0.22	
MGS:igc359	Clostridiales (order)	0.30	
MGS:igc309	*Ruminococcus lactaris*	0.31	
MGS:igc734	*Streptococcus thermophilus*	0.33	
MGS:igc584	*Holdemanella biformis*	0.38	
MGS:igc517	*Faecalibacterium prausnitzii*	0.38	
MGS:igc171	*Faecalibacterium prausnitzii*	0.38	
MGS:igc213	Coprococcus	0.38	
MGS:igc9	*Bacteroides thetaiotaomicron*	0.38	

Table S.2d) below lists the SNPs used in the five GRSs developed in this study. For initial selection of SNPs from other studies, we searched the GWAS Catalog for obesity-related traits (https://www.ebi.ac.uk/gwas/, search key "obesity"). As inclusion criteria for SNPs found in selected studies, we applied a p-value threshold of $p < 10^{-4}$, and we deemed that each study should have more than one SNP present in our dataset in order to be included. This gave us three studies, which fulfilled these criteria and 4-6 SNPs per study. These SNPs all stemmed from case-control setups.

Berndt et al. aimed to investigate utilization of different distribution cut-offs for identification of genetic loci of anthropometric traits with primary focus on BMI. Here, four different clinical classes named overweight (BMI > 25), obesity class I (BMI > 30), obesity class II (BMI > 35) and obesity class III (BMI > 40) were studied for genetic loci influencing BMI. A minimum of 30 cases and 30 controls was required for each study setup.

Bradfield et. al. investigates loci associated with early-onset obesity. Two new SNPs were found with association hereof which both were also found in the wholegrain set. OLFM4 and HOXB5 are involved in cell adhesion and transcription regulation, respectively.

Paternoster et. al. aimed to identify new loci associated with BMI and to determine if extreme sampling designs identifies already known variants. SNPs were found to be involved in apoptosis inhibition (FAIM2), adipose tissue development (FTO), carbohydrate metabolism (GNPDA2), G protein signalling (RGS6), protein transport (SEC16B) and transcription regulation (TFAP2B).

In addition, we performed GWAS on the whole grain cohort to develop two internal GRS. The GWAS in the whole grain study identified non-genome-wide-significant associated SNPs that were included in metabolic pathways such as lipid metabolism (PAK7), adiponectin signalling (CDH13), feeding behaviour (UBE2Q2, ADCY8), PPARγ activity and energy metabolism (FBXO22, PLCB1), brown fat endocrine factors and thermogenesis (NRG4). To limit overfitting from how these SNPs were selected, we only considered the top 10 p-value associated SNPs for two GRS of relative changes in weight and sagittal abdominal diameter during the two intervention periods to build the body weight [kg] and sagittal abdominal diabetes [cm] GRS.

Table S.2d): Genetic risk scores. This table lists the SNPs used for the GRS's, as well as their p-values, effect sizes and the reference from which each was found.

SNP	p-value	Effect size	Phenotype	Paper / GWAS
rs2030323	3e-22	1.12	Obesity (BMI)	Berndt, S. I. et al., "Genome-wide meta-analysis identifies 11 new loci for anthropometric traits and provides insights into genetic architecture," *Nature Genetics*, vol. *45 no. 5*, pp. 501–512, 2013. doi: 10.1038/ng.2606
rs13130484	4e-28	1.08		
rs4735692	3.51e-10	1.04		
rs7138803	1e-20	1.09		
rs4833407	1e-6	1.11	Childhood obesity (BMI)	Bradfield, J. P. et al. (Early Growth Genetics Consortium), "A genome-wide association
rs4864201	2e-7	1.12		

rs9299	4e-9	1.14		meta-analysis identifies new childhood obesity loci," *Nature Genetics*, vol. *44 no.* 5, pp. 526–531, 2012. doi: 10.1038/ng.2247
rs9568856	2e-9	1.22		
rs7138803	6.4e-8	1.24	Extremely overweight young adults (BMI)	Paternoster, L. et al., "Genome-Wide Population-Based Association Study of Extremely Overweight Young Adults – The GOYA Study", *PLoS ONE*, vol. *6 no.* 9: e24303, 2011. doi: 10.1371/journal.pone.0024303
rs9936385	1.4e-13	1.35		
rs13130484	1.9e-5	0.85		
rs699363	2e-5	0.21		
rs543874	6.5e-5	1.2		
rs734597	2e-5	1.25		
rs6077585	3.52e-7	-1.28	Body weight [kg]	GWAS of weight changes in whole grain study
rs1039547	1.59e-6	-2.04		
rs2648435	1.59e-6	-2.04		
rs8036952	1.59e-6	-2.04		
rs7169122	4.68e-6	-1.82		
rs6591079	7.48e-6	-1.27		
rs2239985	9.18e-6	-0.92		
rs13379337	9.79e-6	-0.91		
rs12972098	1.34e-5	-0.91		
rs1015092	1.39e-5	-2.89		
rs17382342	5.74e-6	1.39	Sagittal abdominal diameter [cm]	GWAS of sagittal abdominal diameter changes in whole grain study
rs12156272	6.12e-6	-1.77		
rs10957603	1.47e-6	2.1		
rs536995	1.65e-5	-1.47		
rs17466747	1.7e-5	1.39		
rs4782784	2.17e-5	-1.51		
rs11725412	2.17e-5	-2.26		
exm1537540	2.21e-5	-2.77		
exm1537642	2.21e-5	-2.77		
rs12654643	2.7e-5	-1.8		

Supplementary Material 3: Comparison of models

Table S.3 below is an extended version of Table 2. Here, all models trained and tested on the common set of 130 individuals with complete data across 18 out of 22 datasets are shown, meaning that models which were subsequently not selected for further analysis because of lower performance or suspicion of overfit are included.

The metagenomic data, which was mapped against the MGmapper database, gave five datasets from mapping against catalogues Bacteria, Bacteria draft and Human Microbiome: *Diet.MGm* (contains pre-selected butyrate-producing species for all selected catalogues), *Diet.MGm_A* (contains pre-selected butyrate-producing species for the Bacteria catalogue), *Diet.MGm_B* (contains all species mapped to the Bacteria draft catalogue), *Diet.MGm_B1* (contains pre-selected butyrate-producing species for the Bacteria draft catalogue) and *Diet.MGm_C* (contains pre-selected butyrate-producing species for the Human Microbiome catalogue). The dataset for Bacteria draft catalogue with butyrate-producing species (MGm_B1) was selected for subsequent models, since it performed much better when considering all available data, not just 130 samples, as seen in Table S.6. Furthermore, the model including forward selected species from the Bacteria draft catalogue (MGm_B) showed less separation when considering the difference between models trained on a true and a permuted target.

Table S.3: **Test performances for models run on a common set of 130 individuals with complete data across 50 five-fold shuffle-split cross-validations**. The blue-red colorbar is for area under the receiver operating characteristic curve (ROC-AUC), sensitivity and specificity, while the blue.yellow-red colorbar is for Matthews correlation coefficient (MCC). Abbreviations for model combinations are explained in Table 1.

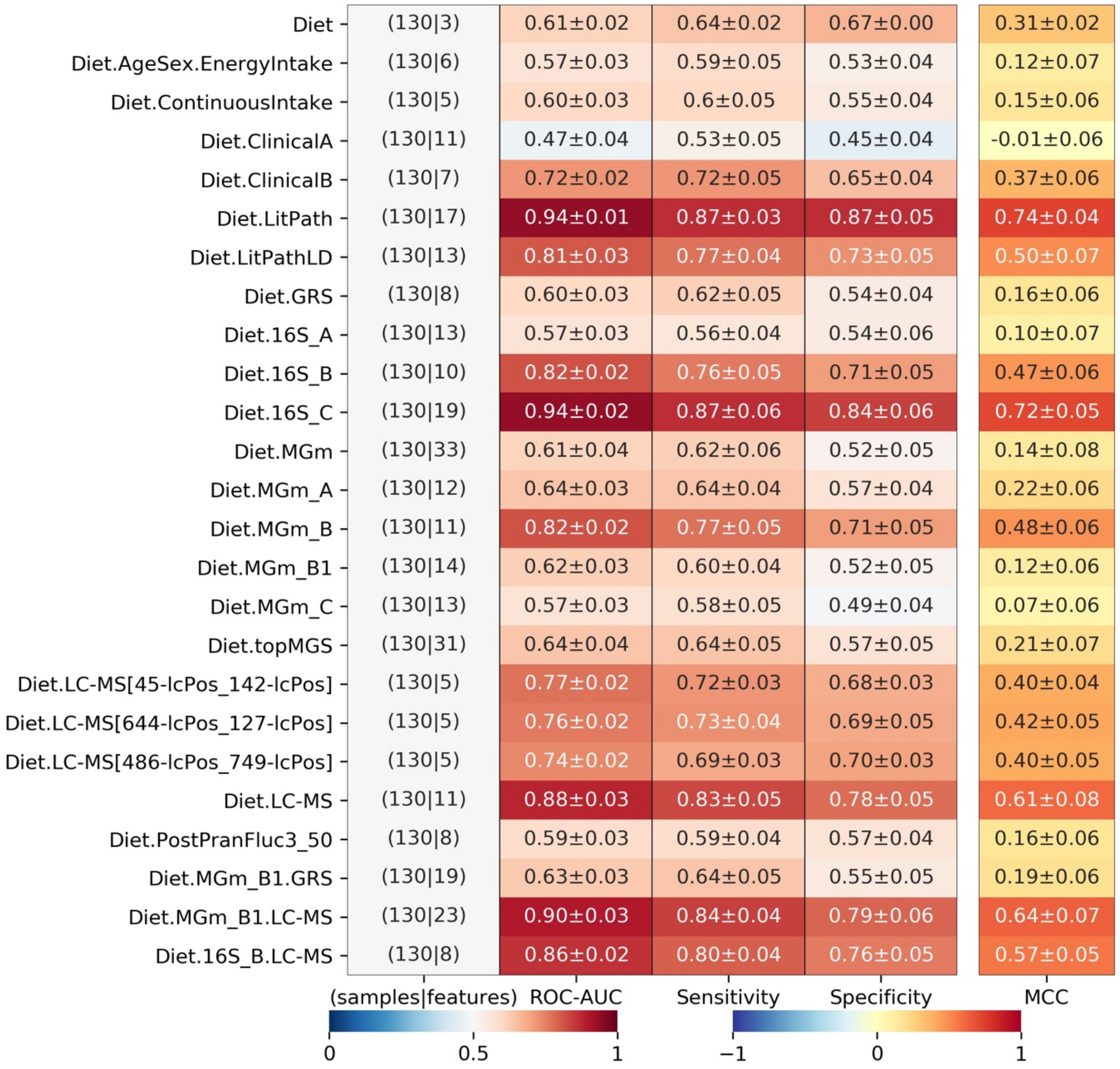
Diet | (130|3) | 0.61±0.02 | 0.64±0.02 | 0.67±0.00 | 0.31±0.02
Diet.AgeSex.EnergyIntake | (130|6) | 0.57±0.03 | 0.59±0.05 | 0.53±0.04 | 0.12±0.07
Diet.ContinuousIntake | (130|5) | 0.60±0.03 | 0.6±0.05 | 0.55±0.04 | 0.15±0.06
Diet.ClinicalA | (130|11) | 0.47±0.04 | 0.53±0.05 | 0.45±0.04 | -0.01±0.06
Diet.ClinicalB | (130|7) | 0.72±0.02 | 0.72±0.05 | 0.65±0.04 | 0.37±0.06
Diet.LitPath | (130|17) | 0.94±0.01 | 0.87±0.03 | 0.87±0.05 | 0.74±0.04
Diet.LitPathLD | (130|13) | 0.81±0.03 | 0.77±0.04 | 0.73±0.05 | 0.50±0.07
Diet.GRS | (130|8) | 0.60±0.03 | 0.62±0.05 | 0.54±0.04 | 0.16±0.06
Diet.16S_A | (130|13) | 0.57±0.03 | 0.56±0.04 | 0.54±0.06 | 0.10±0.07
Diet.16S_B | (130|10) | 0.82±0.02 | 0.76±0.05 | 0.71±0.05 | 0.47±0.06
Diet.16S_C | (130|19) | 0.94±0.02 | 0.87±0.06 | 0.84±0.06 | 0.72±0.05
Diet.MGm | (130|33) | 0.61±0.04 | 0.62±0.06 | 0.52±0.05 | 0.14±0.08
Diet.MGm_A | (130|12) | 0.64±0.03 | 0.64±0.04 | 0.57±0.04 | 0.22±0.06
Diet.MGm_B | (130|11) | 0.82±0.02 | 0.77±0.05 | 0.71±0.05 | 0.48±0.06
Diet.MGm_B1 | (130|14) | 0.62±0.03 | 0.60±0.04 | 0.52±0.05 | 0.12±0.06
Diet.MGm_C | (130|13) | 0.57±0.03 | 0.58±0.05 | 0.49±0.04 | 0.07±0.06
Diet.topMGS | (130|31) | 0.64±0.04 | 0.64±0.05 | 0.57±0.05 | 0.21±0.07
Diet.LC-MS[45-lcPos_142-lcPos] | (130|5) | 0.77±0.02 | 0.72±0.03 | 0.68±0.03 | 0.40±0.04
Diet.LC-MS[644-lcPos_127-lcPos] | (130|5) | 0.76±0.02 | 0.73±0.04 | 0.69±0.05 | 0.42±0.05
Diet.LC-MS[486-lcPos_749-lcPos] | (130|5) | 0.74±0.02 | 0.69±0.03 | 0.70±0.03 | 0.40±0.05
Diet.LC-MS | (130|11) | 0.88±0.03 | 0.83±0.05 | 0.78±0.05 | 0.61±0.08
Diet.PostPranFluc3_50 | (130|8) | 0.59±0.03 | 0.59±0.04 | 0.57±0.04 | 0.16±0.06
Diet.MGm_B1.GRS | (130|19) | 0.63±0.03 | 0.64±0.05 | 0.55±0.05 | 0.19±0.06
Diet.MGm_B1.LC-MS | (130|23) | 0.90±0.03 | 0.84±0.04 | 0.79±0.06 | 0.64±0.07
Diet.16S_B.LC-MS | (130|8) | 0.86±0.02 | 0.80±0.04 | 0.76±0.05 | 0.57±0.05
(samples|features)
ROC-AUC
Sensitivity
Specificity
MCC
0
0.5
1
−1
0
1

Supplementary Material 4: Permuted target distributions of best models with diet, 16S-based OTUs or MGmapped gut microbiome taxa with urine metabolites identified by LC-MS

The robustness of machine learning models, including those trained using small datasets, can be assessed by permutation testing of the prediction label across multiple model initialization. By comparing the random performance to the true performance, it is possible to statistically compare and determine if the learnings given the true label are better than random. This can help identify if the model learned a true signal or noise. Permutation testing is also a great tool on larger datasets, however, it is not often seen in disease machine learning publications, where the focus often is on validation.

Three approaches with the randomly permuted target where used to assess the robustness of our models compared to random. The first approach trained and tested the model on the randomly permuted target, but with the features selected by the true model ($p=7.06*10^{-18}$ and $p=1.51*10^{-47}$, Figure S.5A). The second approach was with the model trained and with feature selection, whereby the model optimized towards the random target ($p=8.86*10^{-10}$ and $p=2.58*10^{-9}$, Figure S.5B). For the third approach, the true model was stored and used to predict on the permuted target ($p=7.06*10^{-18}$ and $p=1.42*10^{-62}$, Figure S.5C).

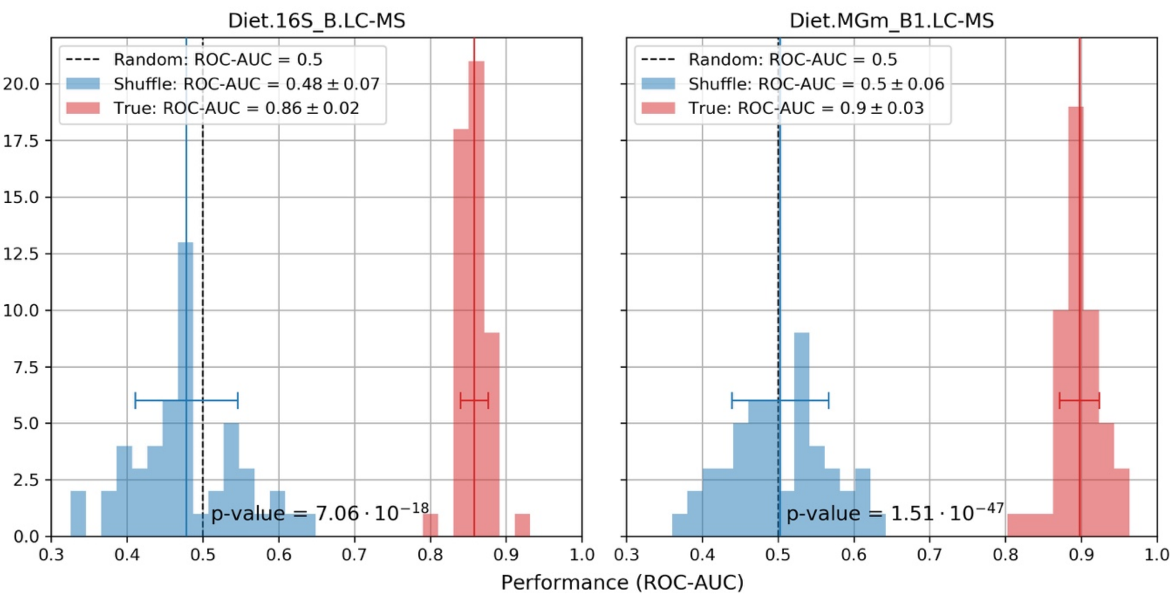

Figure S.5A: Permutation tests. ROC-AUC distributions for two best models with data combinations diet, forward selected 16S-based OTUs (left, Diet.16S_B.LC-MS) or butyrate-producing species (right, Diet.MGm_B1.LC-MS) and forward selected urine metabolites identified by LC-MS trained on 130 individuals with complete data. The features selected by the model trained on the true target (red, true) were kept. The prediction labels were shuffled in the dataset, and models were retrained on the shuffled dataset with the retained features from the true model (blue, shuffle). The black dashed lines denote random ROC-AUC performance of 0.5, while the red and blue lines are the performance means and standard deviations of the model performances.

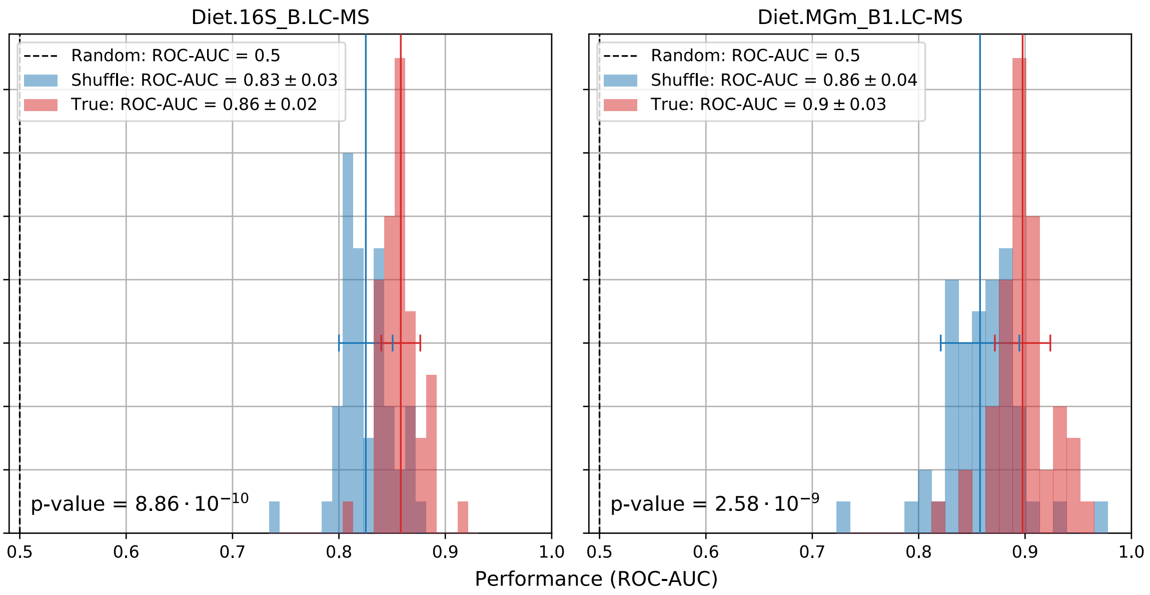

Figure S.5B: Permutation tests. ROC-AUC distributions for two best models with data combinations diet, forward selected 16S-based OTUs (left, Diet.16S_B.LC-MS) or butyrate-producing species (right, Diet.MGm_B1.LC-MS) and forward selected urine metabolites identified by LC-MS trained on 130 individuals with complete data. These were used to train models on the true target (red) and on permuted targets (blue), respectively. The black dashed lines denote random ROC-AUC performance of 0.5, while the red and blue lines are the performance means and standard deviations of the model performances. The prediction labels were shuffled (shuffle) in the dataset, and models were retrained on

the shuffled dataset allowing to model to select new features. The performance was evaluated on the model trained and tested on the shuffled dataset.

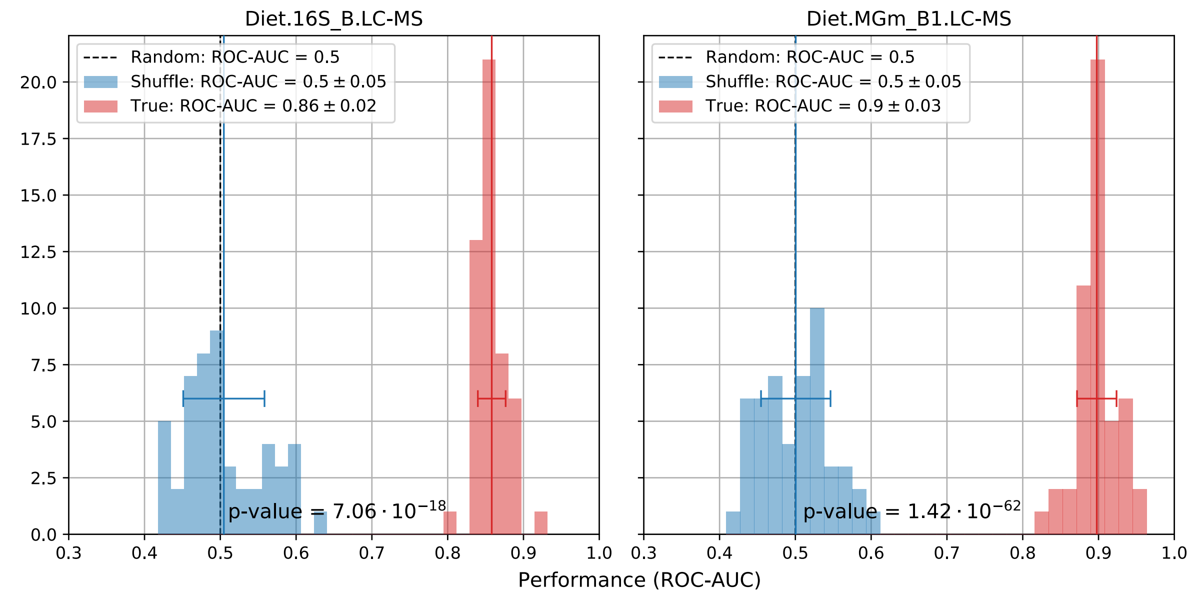

Figure S.5C: Permutation tests. ROC-AUC distributions for two best models with data combinations diet, forward selected 16S-based OTUs (left, Diet.16S_B.LC-MS) or butyrate-producing species (right, Diet.MGm_B1.LC-MS) and forward selected urine metabolites identified by LC-MS trained on 130 individuals with complete data. The model trained on the true target (red, true) was stored, the prediction labels were shuffled (blue, shuffle) in the dataset, and performance was evaluated on the fixed trained model when predicting on the shuffled target data. The black dashed lines denote random ROC-AUC performance of 0.5, while the red and blue lines are the performance means and standard deviations of the model performances.

Supplementary Material 5: Performance of models trained with all samples available for a data combination

Table S.4 below is an extended version of Table 3 and shows the model for each data combination run on the full intersection of samples, i.e. all samples available for a given feature combination. The bold text marks data combinations included in the ensemble model.

Multiple representations for the postprandial features were made, which can be seen in comparison in Table S.4 below, where models have been run with each representation along with the diet features. As seen in the figure, the new representation using the clustered sum of consecutive ones (Diet.PostPranFluc3_50) performs slightly better, which is why this representation was used in subsequent model combinations.

Table S.4: **Test performances for all feature combinations, where models are run on all data available individuals for the combined data types**. The model names in bold are included in the model ensemble. The blue-red colorbar is for area under the receiver operating characteristic curve (ROC-AUC), sensitivity and specificity, while the blue.yellow-red colorbar is for Matthews correlation coefficient (MCC). Abbreviations for model combinations are explained in Table 1.

	(samples|features)	ROC-AUC	Sensitivity	Specificity	MCC
Diet	(203|3)	0.62±0.01	0.64±0.00	0.66±0.00	0.30±0.00
Diet.AgeSex.EnergyIntake	(201|6)	0.65±0.02	0.67±0.03	0.58±0.03	0.25±0.05
Diet.ContinuousIntake	(201|5)	0.63±0.02	0.63±0.03	0.55±0.04	0.18±0.05
Diet.AgeSex.VAS	(147|21)	0.56±0.04	0.54±0.05	0.57±0.05	0.11±0.06
Diet.ClinicalA	(196|11)	0.57±0.02	0.60±0.03	0.54±0.03	0.14±0.04
**Diet.ClinicalB**	(196|7)	0.72±0.02	0.67±0.03	0.67±0.04	0.34±0.04
**Diet.TransitTime**	(195|4)	0.65±0.02	0.67±0.03	0.58±0.02	0.25±0.04
Diet.LitPath	(185|21)	0.93±0.01	0.90±0.02	0.77±0.05	0.68±0.05
**Diet.LitPathLD**	(185|14)	0.77±0.02	0.77±0.04	0.65±0.05	0.42±0.05
Diet.GRS	(185|8)	0.60±0.02	0.66±0.03	0.48±0.04	0.14±0.05
Diet.16S_A	(179|13)	0.51±0.03	0.53±0.03	0.51±0.04	0.04±0.05
Diet.16S_B	(179|14)	0.81±0.02	0.74±0.04	0.73±0.03	0.47±0.04
Diet.16S_C	(179|28)	0.94±0.01	0.87±0.04	0.85±0.04	0.73±0.05
Diet.MGm	(181|33)	0.64±0.03	0.62±0.05	0.59±0.04	0.21±0.05
Diet.MGm_B1	(183|14)	0.67±0.02	0.64±0.04	0.61±0.03	0.25±0.05
Diet.MGm_A	(181|12)	0.58±0.03	0.56±0.05	0.56±0.04	0.12±0.06
Diet.MGm_B	(183|12)	0.80±0.02	0.74±0.04	0.72±0.04	0.46±0.05
Diet.MGm_C	(183|13)	0.56±0.03	0.57±0.04	0.51±0.04	0.09±0.05
Diet.MGS	(185|14)	0.85±0.02	0.79±0.05	0.74±0.05	0.54±0.04
Diet.topMGS	(185|31)	0.61±0.03	0.60±0.04	0.57±0.04	0.17±0.06
Diet.GC-MS	(193|8)	0.80±0.05	0.76±0.06	0.70±0.05	0.46±0.09
**Diet.LC-MS[644-lcPos_127-lcPos]**	(193|5)	0.77±0.02	0.73±0.02	0.69±0.04	0.42±0.04
Diet.LC-MS[1086-lcPos_401-lcPos]	(193|5)	0.76±0.02	0.70±0.03	0.68±0.04	0.38±0.05
Diet.LC-MS[284-lcPos_104-lcPos]	(193|5)	0.74±0.02	0.71±0.03	0.67±0.03	0.38±0.04
Diet.LC-MS	(193|12)	0.84±0.02	0.80±0.04	0.74±0.04	0.54±0.05
Diet.PostPranAUC	(182|8)	0.57±0.03	0.57±0.04	0.55±0.04	0.13±0.05
Diet.PostPranFluc1_10	(203|8)	0.61±0.02	0.62±0.04	0.53±0.03	0.15±0.05
Diet.PostPranFluc2_10	(203|8)	0.62±0.02	0.61±0.03	0.57±0.03	0.18±0.04
Diet.PostPranFluc3_10	(203|8)	0.62±0.02	0.60±0.03	0.58±0.04	0.18±0.05
Diet.PostPranFluc1_50	(203|8)	0.61±0.02	0.62±0.04	0.53±0.03	0.15±0.05
Diet.PostPranFluc2_50	(203|8)	0.63±0.02	0.63±0.04	0.57±0.03	0.20±0.05
**Diet.PostPranFluc3_50**	(203|8)	0.64±0.02	0.63±0.04	0.56±0.03	0.19±0.05
Diet.MGm_B1.GRS	(165|19)	0.67±0.02	0.68±0.04	0.59±0.03	0.27±0.05
Diet.Clinical.GRS	(179|16)	0.57±0.02	0.62±0.03	0.47±0.04	0.10±0.04
Diet.ClinicalA.TransitTime	(188|12)	0.59±0.02	0.61±0.03	0.54±0.03	0.14±0.04
Diet.ClinicalA.TransitTime.GRS	(171|17)	0.58±0.03	0.64±0.04	0.48±0.04	0.12±0.05
Diet.ClinicalA.MGm_A	(175|21)	0.57±0.03	0.56±0.04	0.54±0.04	0.1±0.06
Diet.ClinicalA.MGm_B1	(177|22)	0.68±0.03	0.65±0.04	0.63±0.04	0.27±0.06
Diet.ClinicalA.MGm_C	(177|21)	0.56±0.03	0.56±0.05	0.54±0.04	0.10±0.07
Diet.MGm_B1.TransitTime	(175|15)	0.63±0.03	0.62±0.04	0.59±0.03	0.21±0.05
Diet.MGm_B1.TransitTime.GRS	(157|20)	0.64±0.03	0.66±0.04	0.56±0.04	0.22±0.06
**Diet.MGm_B1.LC-MS**	(173|22)	0.88±0.02	0.83±0.04	0.78±0.04	0.62±0.05
**Diet.16S_B.LC-MS**	(169|8)	0.84±0.02	0.78±0.03	0.74±0.04	0.52±0.04
Diet.MGm_B1.LC-MS.EnergyIntake	(171|24)	0.88±0.02	0.83±0.04	0.79±0.04	0.61±0.05
Diet.16S_B.LC-MS.EnergyIntake	(167|8)	0.81±0.02	0.77±0.04	0.73±0.03	0.5±0.04
Diet.MGm_B1.LC-MS.TransitTime	(171|24)	0.88±0.03	0.82±0.05	0.79±0.04	0.61±0.06
Diet.16S_B.LC-MS.TransitTime	(167|8)	0.82±0.02	0.77±0.03	0.73±0.03	0.50±0.04

Supplementary Material 6: Feature levels by class for microbiome and metabolome

Table S.5: **Feature levels by class for microbiome and metabolomic** features (mean ± standard deviation) if considered very important by the relative Gini coefficient for the number of selected features in Figure 3. The microbiome features are abundances and the metabolites are noted as the logarithm of the relative abundance. NA: not available.

Features	m/z	Retention time	Prevalence (microbiome) or annotation (metabolite)	Prevalence Responders	Prevalence Non responders	Responders	Non responders	p_value of single feature association
**3-lcPos**	82.02	32.55	NA	NA	NA	-3.82 ± 0.18	-3.85 ± 0.19	0.77
**701-lcPos**	265.14	323.54	gamma-carboxyethyl hydroxychroman	NA	NA	-3.62 ± 0.16	-3.59 ± 0.19	0.17
**241-lcPos**	169.10	61.54	NA	NA	NA	-3.93 ± 0.1	-3.95 ± 0.12	0.39
**142-lcPos**	145.10	325.59	food additive (S1)-Methoxy-3-haptanethiol	NA	NA	-3.78 ± 0.12	-3.8 ± 0.11	0.38
**624-lcPos**	249.07	40.12	Dipeptide	NA	NA	-3.92 ± 0.09	-3.93 ± 0.09	0.09
**921-lcPos**	326.04	216.69	Urothion	NA	NA	-3.63 ± 0.12	-3.63 ± 0.11	0.96
**1180-lcPos**	567.18	246.85	NA	NA	NA	-3.91 ± 0.13	-3.94 ± 0.16	0.37
**131-lcNeg**	216.92	37.63	NA	NA	NA	-2.85 ± 0.22	-2.83 ± 0.21	0.85
**539-lcPos**	230.19	81.67	NA	NA	NA	-3.85 ± 0.12	-3.86 ± 0.14	0.44
**470-lcPos**	219.03	36.54	Polyol OR sugar metabolite	NA	NA	-3.62 ± 0.22	-3.65 ± 0.15	0.29
**763-lcPos**	280.05	216.36	3-(3,5-dihydroxyphenyl)-1-propanoic acid sulphate OR Dihydrocaffeic acid 3-sulfate OR similar	NA	NA	-4.08 ± 0.22	-4.05 ± 0.22	0.44
**557-lcPos**	235.09	41.67	Dipeptide	NA	NA	-3.68 ± 0.1	-3.71 ± 0.11	0.1
**918-lcPo**	325.03	37.20	NA	NA	NA	-3.71 ± 0.31	-3.65 ± 0.21	0.55
**680-lcPos**	262.04	94.03	2-Methoxyacetaminophen sulfate OR p-Coumaric acid sulfate	NA	NA	-2.49 ± 0.09	-2.48 ± 0.08	0.46
**749-lcPos**	276.99	451.41	NA	NA	NA	-4.18 ± 0.1	-4.19 ± 0.09	0.37
**1182-lcPos**	577.13	240.86	NA	NA	NA	-4.05 ± 0.17	-4.07 ± 0.15	0.42
**486-lcPos**	221.08	38.04	dipeptide L-β-aspartypl-L-serine/Aspartyl-Serine	NA	NA	-4 ± 0.18	-4 ± 0.15	0.5
Ruminococcaceae.	NA	NA	97/169	47/89	50/80	4.74e-03±9.31e-03	3.74e-03±6.03e-03	0.32
Streptococcus. sp	NA	NA	60/169	36/89	24/80	3.50e-03±1.22e-02	4.74e-03±1.81e-02	0.46
F. prausnitzii	NA	NA	172/173	88/89	84/84	9.46e-03±5.33e-03	1.32e-02±9.07e-03	0.01
E. ramulus	NA	NA	173/173	89/89	84/84	7.34e-02±6.28e-02	7.21e-02±5.30e-02	0.41
R. faecis	NA	NA	173/173	89/89	84/84	8.70e-01±1.14e+00	6.60e-01±7.70e-01	0.25
R. intestinalis	NA	NA	124/173	68/89	56/84	7.98e-04±4.81e-04	6.79e-04±4.95e-04	0.12
R. inulinivorans	NA	NA	149/173	79/89	70/84	9.55e-04±4.24e-04	9.52e-04±5.58e-04	0.82
B. fibrisolvens	NA	NA	15/173	5/89	10/84	1.12e-04±5.53e-04	1.90e-04±6.67e-04	0.15
S. variabile	NA	NA	144/173	76/89	68/84	1.24e-03±8.26e-04	1.19e-03±8.98e-04	0.53
A. colihominis	NA	NA	91/173	48/89	43/84	5.62e-04±5.43e-04	5.24e-04±5.26e-04	0.67
B. uniformis	NA	NA	98/173	53/89	45/84	1.07e-03±1.46e-03	1.36e-03±2.32e-03	0.88
E. hallii	NA	NA	13/173	8/89	5/84	8.99e-05±2.88e-04	7.14e-05±3.02e-04	0.47

Supplementary Material 7: Ensemble scoring performances

Table S.6: **Ensemble performance across scoring methods.** This table shows the performance of the ensemble from models marked in bold in Table 3, when scoring with the four different approaches at prediction score thresholds {s= [≤0.30 or ≥0.70], [≤0.25 or ≥0.75], [≤0.20 or ≥0.80]}. *s* represents a given prediction score. The table is an extension of Figure 4a.

**Scoring method**	**Confidence**	**ROC-AUC**	**Sensitivity**	**Specificity**	**MCC**
Mean	None	0.86	0.73	0.70	0.43
Majority voting	None	0.73	0.76	0.69	0.46
Mean of confident scores	$s \leq 0.30$ or $s \geq 0.70$	0.84	0.69	0.70	0.39
Majority voting on confident scores	$s \leq 0.30$ or $s \geq 0.70$	0.70	0.70	0.70	0.40
Mean of confident scores	$s \leq 0.25$ or $s \geq 0.75$	0.83	0.69	0.70	0.39
Majority voting on confident scores	$s \leq 0.25$ or $s \geq 0.75$	0.69	0.69	0.70	0.39
Mean of confident scores	$s \leq 0.20$ or $s \geq 0.80$	0.82	0.69	0.71	0.40
Majority voting on confident scores	$s \leq 0.20$ or $s \geq 0.80$	0.70	0.69	0.71	0.40
Without microbiome: Mean of confident scores	$s \leq 0.25$ or $s \geq 0.75$	0.72	0.69	0.66	0.35
